# Prognostic biomarker and clinical significance of PLOD gene family in clear cell renal cell carcinoma

**DOI:** 10.3389/fonc.2025.1613540

**Published:** 2025-10-10

**Authors:** Xuan Shang, Liu Liu, Zhenwei Yang, Min Yan, Ruimin Ren, Kexin Guo, Jie Wang, Wei Zhang, Jiasong Chang, Jialei Li, Jimin Cao, Guang Li, Lijuan Gao

**Affiliations:** ^1^ Department of Cardiology, The First Hospital and First College of Clinical Medicine, Shanxi Medical University, Taiyuan, China; ^2^ Key Laboratory of Cellular Physiology at Shanxi Medical University, Ministry of Education, Taiyuan, China; ^3^ Department of Physiology, Shanxi Medical University, Taiyuan, China; ^4^ Department of Urology, Shanxi Bethune Hospital (Third Hospital of Shanxi Medical University), Taiyuan, China; ^5^ Department of Urology, The First Hospital and First College of Clinical Medicine, Shanxi Medical University, Taiyuan, China; ^6^ Department of Cardiology, The Affiliated Hospital of Southwest Medical University and Key Laboratory of Medical Electrophysiology, Ministry of Education & Institute of Cardiovascular Research, Southwest Medical University, Luzhou, Sichuan, China

**Keywords:** clear cell renal cell carcinoma (ccRCC), PLOD gene family, prognostic biomarker, collagen biosynthesis, bioinformatics

## Abstract

**Background:**

The PLOD gene family, involved in extracellular matrix (ECM) remodeling, plays a role in tumor progression, but its comprehensive role and clinical significance in clear cell renal cell carcinoma (ccRCC) remains unclear.

**Methods:**

We integrated multi-omics bioinformatics analyses from public databases (TCGA, GEO) with experimental validation using RT-qPCR, western blotting, and functional assays to systematically evaluate the expression patterns, prognostic value, immune microenvironment associations and drug resistance of PLOD genes in ccRCC. Computational approaches, including the comparative toxicogenomics database and molecular docking, were further employed to identify potential chemical modulators.

**Results:**

*PLOD1*, *PLOD2*, and *PLOD3* were consistently overexpressed at both mRNA and protein levels in ccRCC tissues and cell lines. High PLOD expression was significantly correlated with reduced overall survival, and poor disease-free survival. Functional enrichment analysis revealed the involvement of PLOD gene family in collagen biosynthesis, ECM-receptor interaction, and lysine degradation pathways. PLOD expression was also linked to an immunosuppressive microenvironment and resistance to conventional therapeutics. Through toxicogenomics screening and molecular docking, acetaminophen was identified as a potential regulator of all three PLOD proteins.

**Conclusions:**

This study underscores the pivotal role of the PLOD family in ccRCC pathogenesis through ECM remodeling, immune modulation, and therapy resistance. Our results support their utility as diagnostic and prognostic biomarkers, and acetaminophen may serve as a candidate for targeting PLOD-mediated pathways, providing a foundation for future preclinical and therapeutic investigations.

## Introduction

Renal cell carcinoma (RCC), representing 2%−3% of all malignant tumors, is a prevalent urological malignancy with significant clinical impact (1–3). Among its histological subtypes, clear cell renal cell carcinoma (ccRCC) predominates, accounting for 75%−80% of RCC cases (4–6). Notably, the incidence and mortality rates of ccRCC have shown an alarming upward trend in recent years, with a concerning shift toward younger age groups. While surgical intervention remains the cornerstone of treatment for localized ccRCC, the management of metastatic disease continues to pose substantial therapeutic challenges, often resulting in poor clinical outcomes (1). As a genetically driven malignancy, ccRCC has been the focus of extensive molecular investigations. Despite significant advancements in molecular biology techniques that have enabled the identification of numerous genes associated with ccRCC pathogenesis, the precise molecular mechanisms underlying tumor development and metastasis remain elusive (7–9). This knowledge gap underscores the critical need for identifying novel diagnostic biomarkers and therapeutic targets to improve patient outcomes.

Recent insights into tumor biology have highlighted the crucial role of extracellular matrix (ECM) remodeling in cancer progression (10). In ccRCC, tumor progression is characterized by extensive collagen deposition and ECM stiffening, processes critically dependent on procollagen-lysine 2-oxoglutarate 5-dioxygenase (PLOD)-mediated collagen cross-linking modifications. The PLOD family, comprising *PLOD1*, *PLOD2*, and *PLOD3*, regulates collagen biosynthesis through lysyl hydroxylation, serving as a central regulator of ECM remodeling (11). Emerging evidence demonstrates the oncogenic potential of PLOD family members across various malignancies (12): *PLOD1* promotes osteosarcoma proliferation and metastasis while correlating with clinical features in breast cancer (13, 14); *PLOD2* facilitates immune evasion in sarcoma and enhances breast cancer cell proliferation (14); and *PLOD3* drives colorectal cancer liver metastasis through vascular microenvironment remodeling (15). These findings collectively position the PLOD family as a potential pan-cancer therapeutic target.

The PLOD gene family, encoding lysyl hydroxylases critical for collagen biosynthesis, plays a pivotal role in ECM remodeling through lysyl hydroxylation. In ccRCC, extensive ECM remodeling and abnormal angiogenesis are hallmark features, with PLOD-driven collagen modification serving as key regulators of these processes (16). The hypoxic tumor microenvironment characteristic of ccRCC may further amplify PLOD activity, as hypoxia-inducible factor (HIF) signaling pathways are known to specifically activate PLOD expression, potentially creating a therapeutic vulnerability (17). A foundation study by Xu et al. (18) established that PLOD gene family are significantly upregulated in ccRCC tissues, and their elevated mRNA expression is associated with advanced tumor stage and poor patient survival. While this seminal work identified PLODs as promising prognostic biomarkers, their precise roles in modulating the tumor immune microenvironment, regulating therapeutic response, and the potential for pharmacological targeting remain largely unexplored. Therefore, to build upon these initial finding, our study aims to provide a comprehensive analysis of the immunological functions and therapeutic vulnerabilities associated with PLOD gene family in ccRCC.

This study systematically investigates the expression patterns, prognostic value, immune microenvironment interactions, and therapeutic targeting potential of the PLOD family in ccRCC, aiming to elucidate its molecular mechanisms in driving tumor progression and provide theoretical foundations for developing novel diagnostic and therapeutic strategies. Through integrated bioinformatic analysis and functional validation, we comprehensively characterize the biological functions and clinical translation potential of the PLOD family in ccRCC for the first time, offering new perspectives for improving patient outcomes.

## Materials and methods

### GEPIA analysis

GEPIA (19) (http://gepia.cancer-pku.cn/) was used to analyze the expression profiles of PLODs acquired through pan-cancer analysis. The study included 523 ccRCC tissue samples and 100 normal kidney tissue samples. The expressions of PLODs in ccRCC tissues and normal renal tissues were compared using GEPIA2. mRNA data were acquired from the Cancer Genome Atlas (TCGA) and Genotypic Tissue Expression (GTEx) databases to visualize the transcriptional profiles of PLODs. Cut off: |Log2FC|=1, q-value=0.01.

### UALCAN dataset analysis

UALCAN (20) (http://ualcan.path.uab.edu/) is a comprehensive tool that provides analysis according to TCGA dataset. The study included 533 ccRCC tissue samples and 72 normal kidney tissue samples. We analyzed the transcriptional levels of PLOD genes between ccRCC tissues and adjacent normal tissues. The relationship between the expression levels of PLOD genes and tumor stage was also analyzed using this database. *P* < 0.05 set as the cut off.

### Cell culture

The cell lines used in this study were obtained from commercial sources: HK-2 was sourced from Procell Life Science & Technology Co., Ltd., while A498 and 786-O were acquired from the American Type Culture Collection (ATCC). HK-2 cells were maintained in DMEM/F12, 786-O cells in RPMI 1640, and A498 cells in MEM. All cell cultures were grown at 37 °C in a humidified incubator with 5% CO_2_ and supplemented with 10% fetal bovine serum (FBS).

### Patients and kidney tissue sampling

The study included eight patients diagnosed as ccRCC in Shanxi Bethune Hospital, Taiyuan, China. The institutional ethics approval was authorized by Shanxi Bethune Hospital and informed written consents were obtained from all patients. The ccRCC tissues and paired adjacent normal kidney tissues were harvested during tumor resection, and were frozen in liquid nitrogen and stored at an ultra-low-temperature freezer before use. Total RNAs and proteins were extracted respectively for RT-qPCR and western blotting to verify the bioinformatic results.

### RNA isolation and quantitative PCR

The mRNA levels of the PLOD family were detected using quantitative PCR (qPCR) in ccRCC cell lines (786-O and A498), normal renal epithelial cells (HK-2), as well as in ccRCC tissues and their paired normal tissues. Total RNA was extracted from tissues using M5 Universal RNA Mini Kit (Mei5bio, Beijing, China) according to the manufacturer’s instruction. qPCR was performed according to the instructions of Vazyme HiScript III RT SuperMix for qPCR (Vazyme, Nanjing, China). Primer sets for selected genes were designed with the assistance of Sangon Biotech Co., Ltd (Shanghai, China). The expression data were normalized to the reference β-actin and the mRNA levels were calculated using the 2^-ΔΔCt^ method. Primer sequences for qPCR were as follows: β-actin forward: 5’-CCTGGCACCCAGCACAAT-3’, β-actin reverse: 5’-GGGCCGGACTCGTCATAC-3’. *PLOD1* forward: 5’- AAGCCGGAGGACAACCTTTTA-3’, *PLOD1* reverse: 5’- GCGAAGAGAATGACCAGATCC-3’. *PLOD2* forward: 5’- GACAGCGTTCTCTTCGTCCTCA-3’, *PLOD2* reverse: 5’- CTCCAGCCTTTTCGTGGTGACT-3’. *PLOD3* forward: 5’- GCGCCAGTGGAAGTACAAGGAT-3’, *PLOD3* reverse: 5’- CACTTCATCTAAAGCCCCGTTGA -3’.

### Western blotting

Western blotting was performed to extract total protein from ccRCC cell lines (786-O and A498), normal renal epithelial cells (HK-2), as well as from ccRCC tissues and their paired adjacent normal tissues. Total proteins for western blotting were extracted from ccRCC tissues and paired adjacent normal tissues. The concentration of all protein samples was determined using the bicinchoninic acid (BCA) assay (Solarbio Co., Ltd, Beijing, China). A total amount of 30 μg extracted proteins of each sample were separated by 10% SDS-PAGE gel. Next, proteins from the SDS-PAGE gel were transferred onto polyvinylidene fluoride (PVDF) membranes (Millipore, Billerica, MA, USA). Membranes were blocked with 5% nonfat milk for 1 h at room temperature. The membranes were incubated with antibody of *PLOD1* (1:1000, Proteintech, 29480-1-AP), *PLOD2* (1:1000, Proteintech, 66342-1-Ig), *PLOD3* (1:1000, Proteintech, 60058,1-Ig) or β-actin (1:5000, Bioworld, AP0060) for overnight at 4 °C. The membranes were washed with TBST and then were incubated with a secondary antibody conjugated with horseradish peroxidase (Zhongshan Golden Bridge Biotechnology, Beijing, China) for 1 h at room temperature, and were washed again with TBST. Finally, ECL luminescence solution (SEVEN, Beijing, China) was added and the blots were scanned using Image Lab™ Touch Software (Bio-Rad Laboratories, Hercules, CA, USA). The gray values of protein bands were determined using ImageJ, and β-actin or GAPDH was used for normalization.

### Human Protein Atlas dataset analysis

The Human Protein Atlas (HPA) (http://www.proteinatlas.org/) was used to analyze the protein expression levels of PLODs in ccRCC tissues and adjacent normal tissues.

### Survival analysis of PLOD gene family in ccRCC

Gene expression profiles and clinical data for ccRCC patients were obtained from TCGA database. Patients were stratified into high and low expression groups based on median PLOD gene expression levels. Tumor grades were classified as Grade 1 to Grade 4 according to standard pathological criteria. Kaplan-Meier (KM) plotter (21) (https://kmplot.com/analysis/) were generated to evaluate the prognostic value of PLOD gene family expression in ccRCC. Overall survival (OS) and disease-free survival (DFS) were analyzed, and the log-rank test was used to compare survival differences between subgroups. Statistical significance was set as *P* < 0.05. To assess the interaction between PLOD genes expression and tumor grade, patients were further stratified into subgroups based on PLOD expression levels (high *vs*. low/medium) and tumor grade (Grade 1 to Grade 4). KM curves were used to visualize survival outcomes, and the log-rank test was applied to determine statistical significance. The analysis was performed using R software (version 4.2.3). The Wilcoxon rank-sum test was used to compare PLOD expression levels across tumor grades.

### Receiver Operating Characteristic analysis

To evaluate the diagnostic potential of PLOD1, PLOD2, and PLOD3 expression in distinguishing ccRCC from normal kidney tissues, we performed ROC curve analysis using mRNA expression data. The “pROC” package in R was used to generate ROC curves for each gene, and the area under the curve (AUC) was calculated to quantify diagnostic accuracy. The 95% confidence interval of AUC, optimal threshold (based on the maximum Youden index), as well as the corresponding sensitivity and specificity were determined.

### Univariate and multivariate Cox regression analysis

To assess the prognostic value of PLOD gene expression and clinical parameters in ccRCC, we conducted univariate and multivariate Cox proportional hazards regression analyses using the “survival” package in R, with OS as the endpoint. The analyses included genes and indicators such as age, gender, T stage, N stage, M stage, and tumor grade. Univariate analysis evaluated each variable individually, while multivariate analysis incorporated all variables simultaneously. Hazard ratios (HR), 95% confidence intervals, and *p*-values were extracted. The results were presented in the form of forest plots, where the distribution of HRs was represented by red dots for risk factors and green dots for protective factors. The proportional hazards assumption was verified using Schoenfeld residuals.

### cBioPortal database analysis

The cBio Cancer Genomics Portal (cBioPortal) (https://www.cbioportal.org/) was used to evaluated the copy number variants (CNV), mutations, and gene types in ccRCC patients. In addition, we analyzed the relationship between gene mutation and ccRCC prognosis using the cBioPortal tool based on TCGA database. DFS and OS were also analyzed for with or without PLODs mutation in kidney renal clear cell carcinoma (KIRC).

### Immune infiltration analysis

The training set of patient data for KIRC, including gene expression profiles and clinical information, was obtained from the TCGA database (https://portal.gdc.cancer.gov/). The relative abundances of various immune cell types were estimated using seven widely recognized algorithms: CIBERSORT, CIBERSORT-ABS, XCELL, EPIC, MCP-COUNTER, quanTIseq, and TIMER. This multi-algorithm approach was adopted to enhance the robustness of the estimates and mitigate bias inherent to any single method. Immune infiltration scores generated by each tool were subsequently correlated with the expression levels of PLOD1, PLOD2, and PLOD3 using Spearman’s rank correlation analysis. Data analysis was performed using R software (version 4.2.3).

### Tumor Immune Single-cell Hub database analysis

TISCH (http://tisch.comp-genomics.org) is a scRNA-seq database dedicated to the tumor microenvironment (TME). TISCH employs a standardized workflow to uniformly process RNA-seq data, effectively eliminating batch effects between samples, consistently annotating cell types, and identifying malignant cells. The online visualization and analysis features of TISCH empower the biomedical research community to investigate gene expression within the TME at single-cell resolution. Two GEO datasets (GSE111360 and GSE139555) were retrieved and analyzed in this investigation.

### TF-target and miRNA-target of PLOD gene family

The target genes of PLODs were predicted using Targetscan (http://www.targetscan.org/) and GeneCards (https://www.genecards.org/). The intersections of predictions from the two databases were matched by FunRich software to ascertain their regulatory targets. Starbase (http://starbase.sysu.edu.cn/) and Targetscan (http://www.targetscan.org/vert_72/) were used to locate the upstream miRNAs.

### Interactions of PLOD genes verified by STRING

STRING (https://string-db.org/) was used to search co-expression genes of PLODs and to conduct protein-protein interaction (PPI) networks with an interaction score >0.4.

### Gene Ontology and Kyoto Encyclopedia of Genes and Genomes pathway enrichment analysis

We performed GO and KEGG analysis to identify the potential biological functions and pathways associated with DEGs. In GO analysis encompasses three main categories: Molecular Function (MF), Cellular Component (CC), and Biological Process (BP). We used “clusterProfiler”, “org.Hs.eg.db”, and “enrichplot” R package to GO and KEGG analysis. The filtering threshold of P value was 0.05.

### GSCA database for drug sensitivity analysis 

GSCA (https://guolab.wchscu.cn/GSCA/#/) is an online analytical tool for genomic, pharmacogenomic, and immunogenomic gene set cancer analysis. By using the Genomics of Drug Sensitivity database (GDSC) and the Therapeutics Response Portal (CTRP), GSCA compiles drug sensitivity data alongside gene expression profiles from cancer cell lines. In this study, we employed both the drug sensitivity data and PLODs expression profiling information pertaining to cancer cell lines.

### Prediction of chemicals interacting with PLOD gene family

We utilized the Comparative Toxicogenomics Database (CTD) to systematically identify chemicals interacting with the PLOD gene family. To ensure biological relevance and high confidence, we focused on chemicals with documented evidence of direct molecular interactions—such as binding, expression regulation, or functional modulation—rather than relying on arbitrary numerical scores. This evidence-based approach allowed us to prioritize robust candidate chemicals for subsequent molecular docking analysis.

### Docking analysis of affinity between chemicals and PLOD gene family

Molecular docking approach was used to identify the affinities of predicted chemicals with *PLOD1*, *PLOD2*, and *PLOD3*. The structures of the chemicals from PubChem database (http://pubchem.ncbi.nlm.nih.gov/) were downloaded, and Chem3D software was used to minimize the ligand molecular energy. The 3D structures of PLOD genes were obtained from PDB database (http://www1.rcsb.org/) or UniProt database (http://www.uniprot.org/). AutoDockTools 1.5.6 software was used to find out the active pockets. Vina script was run to calculate the molecular binding energy, Vina ≤ −7.0 kcal.mol^−1^ indicated strong binding of “ligand” with “receptor”. PyMOL software was used to display the results.

### Cell migration analysis

*PLOD3* small interfering (si)RNA and Control siRNA was transfected into 786-O cell lines using Lipofectamine2000 reagent (Invitrogen) according to the manufacture’s protocol. Migration was detected with a wound healing assay using a 6-well plate. When the confluence of 786-O cells reached ~90-100% in RPMI-1640 medium with 10% FBS (Cellmax, Beijing, China), scratch wounds were created in each cell. After scratching, the debris was removed and FBS free medium was added, the cell images were obtained after 0, 12, 24 h of incubation at 37°C. Migration was calculated as the relative percentage of scratch area to the area at 0 h (% of wound closure) using ImageJ software.

### Statistical analysis

All statistical analyses were performed using Graph-Pad Prism 6.0 software. Data were represented as the mean ± standard error of mean (SEM). Two-tail *t*-test was used to compare the means of two sample groups. Statistical significance was set at *P* < 0.05.

## Results

### Elevated mRNA and protein expression of PLOD gene family in ccRCC

**Figure 1:**
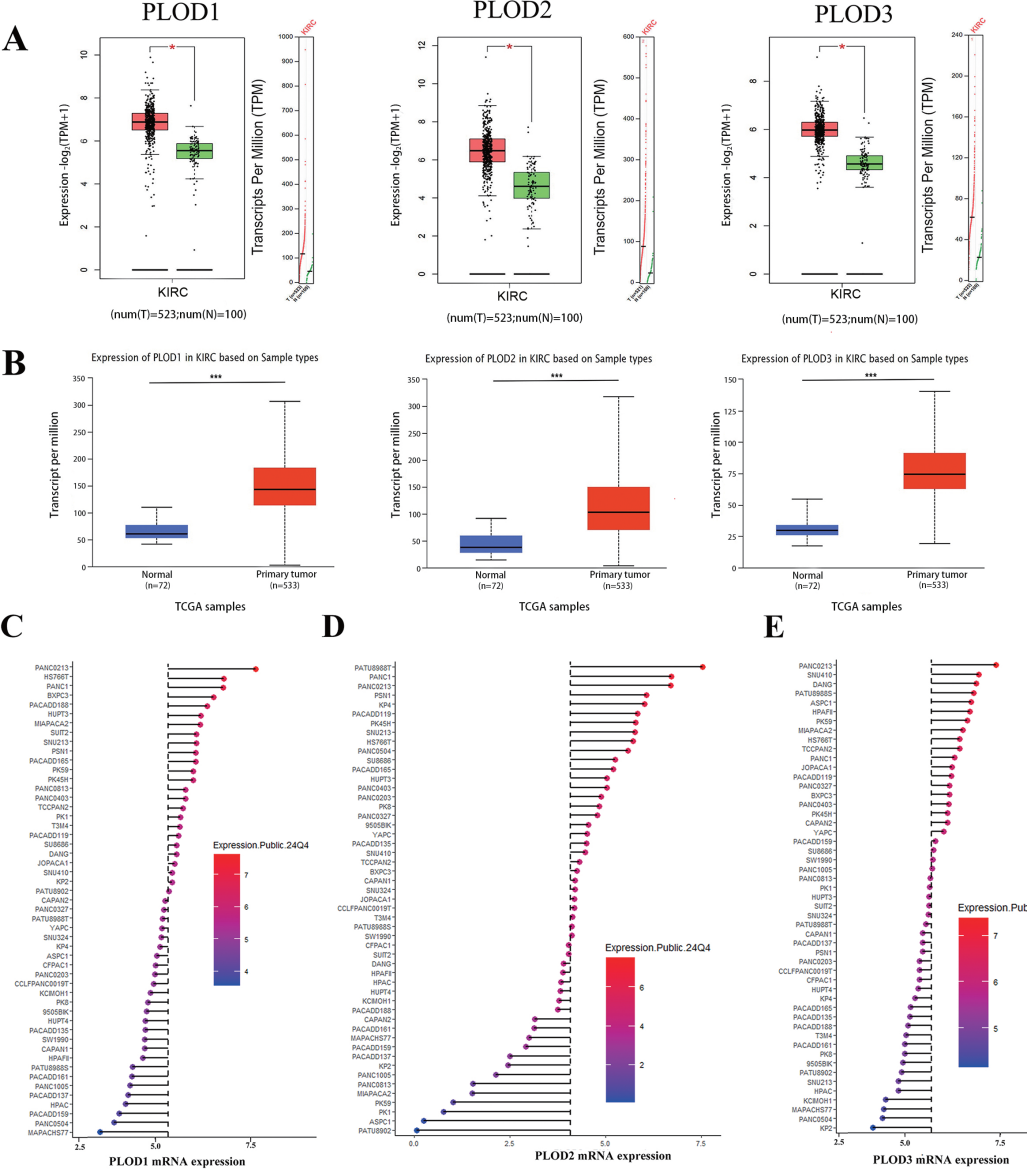
The mRNA expression levels of PLOD gene family in ccRCC tissues and cell lines. **(A)** Boxplot results of mRNA expression levels of *PLOD1*, *PLOD2* and *PLOD3* in ccRCC analyzed using GEPIA. The study included 523 ccRCC tissue samples and 100 normal kidney tissue samples. **(B)** Boxplot of UALCAN database analysis showed the relative mRNA expression of *PLOD1*, *PLOD2* and *PLOD3* in ccRCC tissues compared with adjacent normal kidney tissues. The study included 533 ccRCC tissue sample and 72 normal kidney tissue sample. **(C-E)** mRNA expression of PLOD gene family in various ccRCC cell lines. Blue represents low expression and red represents high expression. The size of the circle represents the amount of expression. *P < 0.05, ***P < 0.001.

In this study, the terms “KIRC”and “ccRCC” are used interchangeably, as both refer to the same entity: clear cell renal cell carcinoma. This nomenclature aligns with standard usage in public databases such as TCGA, where “KIRC” is commonly employed. For clarity and consistency, we have explicitly stated this equivalence throughout the manuscript. To comprehensively characterize the expression profile of PLOD genes in human cancers, we initially analyzed their mRNA levels across multiple cancer types using the TIMER and GEPIA databases. Our analysis revealed significant upregulation of PLOD gene transcripts in various malignancies compared to their corresponding normal tissues, with particularly pronounced overexpression observed in ccRCC (**Supplementary Figures S1AC**). To validate these findings, we employed GEPIA and UALCAN online platforms along with GEO datasets to analyze PLOD expression patterns. Consistent with our initial observations, all three PLOD family members were markedly elevated in ccRCC specimens compared to normal kidney tissues (**Figures 1A, B**; **Supplementary Figure S2**)​. To further investigate the cellular context of PLOD expression, we analyzed ccRCC cell lines using the CCLE database. The results demonstrated consistently high expression levels of all PLOD family members across multiple ccRCC cell lines (**Figures 1C–E**), reinforcing the clinical observations from tissue analyses and supporting the biological relevance of PLOD overexpression in ccRCC pathogenesis.

**Figure 2:**
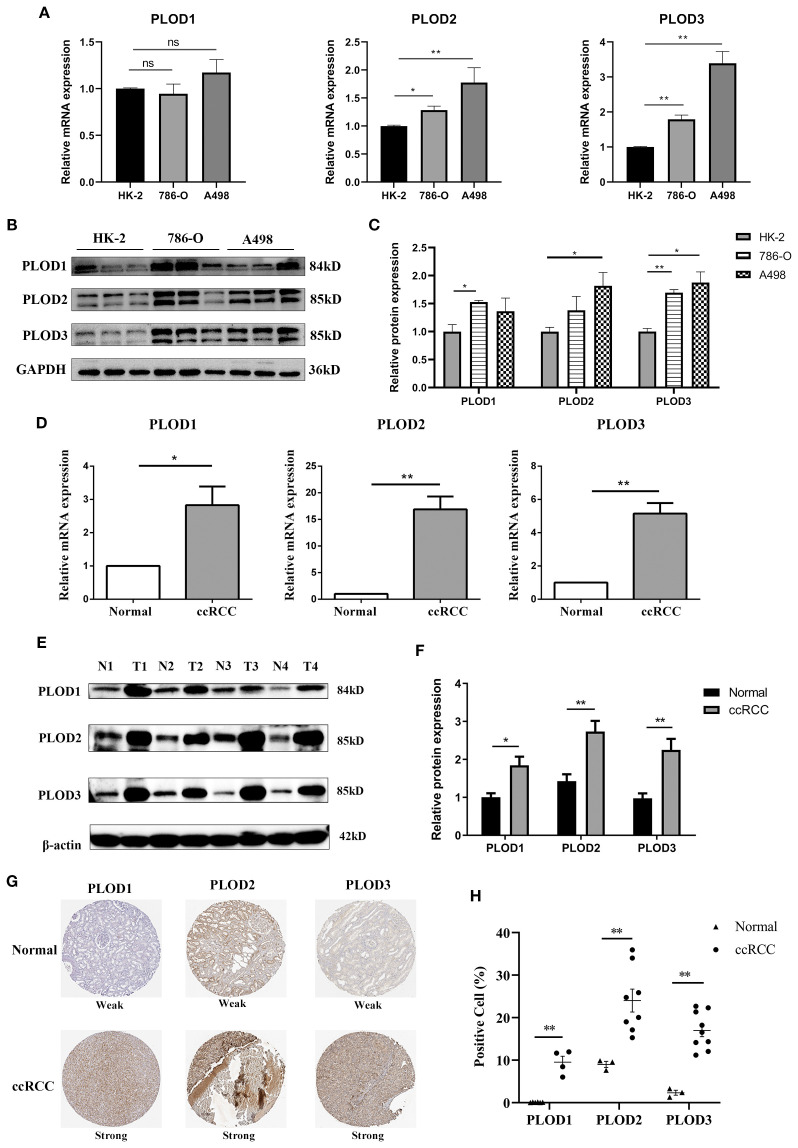
Elevated expression of PLOD gene family in ccRCC cell lines and tissues. **(A)** The mRNA levels of PLOD gene family in normal kidney epithelial cell HK-2 and ccRCC cell lines (A498 and 786-O) detected by qPCR (n=3). **(B)** The protein levels of PLOD gene family in normal kidney epithelial cell HK-2 and ccRCC cell lines (A498 and 786-O) detected by western blotting (n=3). **(C)** The relative protein expression levels of PLOD gene family in **(B)** were analyzed by ImageJ software. **(D)** qPCR analysis showed the relative mRNA levels of PLOD gene family in ccRCC tissues compared with adjacent normal kidney tissues (n=4). **(E)** Western blotting analysis of PLOD gene family expression levels in ccRCC tissues (T) and matched normal kidney tissues (N) (n=4). **(F)** The relative protein expression levels of PLOD gene family in **Figure 3E** were analyzed using ImageJ software. **(G)** Immunohistochemical stains of *PLOD1*, PLO2 and *PLOD3* proteins in ccRCC tissues and normal kidney tissues retrieved from HPA dataset. **(H)** the percentage of positive cells for *PLOD1*, *PLOD2* and *PLOD3* in normal and ccRCC tissues. *P < 0.05, **P < 0.01. ns, non significant difference.

To bridge the gap between bioinformatic predictions and clinical reality, we conducted comprehensive experimental analyses on both ccRCC cell lines (786-O and A498) (22, 23) and patient specimens. Using 8 matched ccRCC/normal tissue pairs (6 T1b and 2 T2 stage; 7 Fuhrman grade 2 and 1 grade 3), we confirmed significant PLOD upregulation at both mRNA (**Figures 2A, D**) and protein levels (**Figures 2B, C, E, F**; **Supplementary Figure S3**) in tumor samples compared to normal controls. These results were further corroborated by immunohistochemical analysis of HPA database samples, which revealed markedly increased PLOD-positive cells in ccRCC tissues (**Figures 2G, H**).

### Prognostic and diagnostic value of PLOD gene family in ccRCC

Having established PLOD overexpression in ccRCC, we next investigated its clinical relevance. Stage-based analysis showed that while PLOD expression levels effectively discriminated tumors from normal tissue across all stage, they showed limited variation among different tumor stages (**Supplementary Figure S4A**). This consistent expression pattern suggests that PLOD levels may serve as a diagnostic indicator for ccRCC, potentially identifying the disease even at stage 1. Furthermore, the similar expression profiles among *PLOD1*, *PLOD2*, and *PLOD3* indicate that all three family members could effectively reflect ccRCC progression.

**Figure 3:**
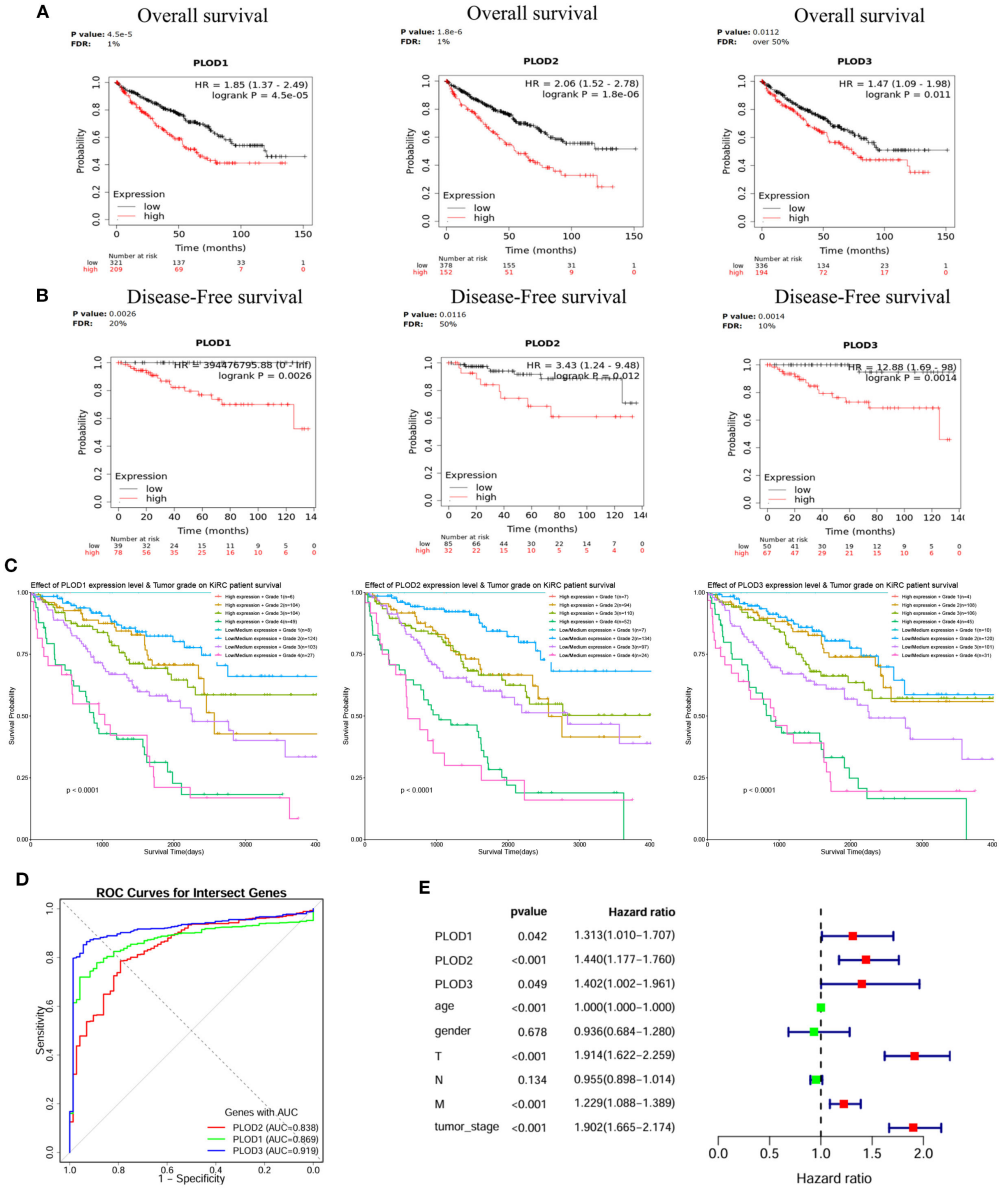
Prognostic significance of PLOD gene family in ccRCC. **(A)** KM curves showing the association between elevated *PLOD1*, *PLOD2*, and *PLOD3* mRNA expression and reduced OS in ccRCC patients. **(B)** KM curves demonstrating the correlation between high PLOD expression levels and worse DFS outcomes. **(C)** Combined effects of PLOD expression and tumor grade on patient survival. Low/medium PLOD expression in low tumor grade (Grade 1/2) is associated with the best survival outcomes, while high PLOD expression in high tumor grade (Grade 3/4) shows the poorest survival outcomes, characterized by a steep decline in survival probability (*P* < 0.0001). **(D)** ROC curves illustrating the diagnostic performance of PLOD gene family for distinguishing ccRCC from normal tissue. AUC values for each gene are indicated. **(E)** Forest plot of univariate Cox regression analysis for PLOD genes and clinicopathological parameters, showing HR with 95% confidence intervals.

Survival analysis using Kaplan-Meier curves demonstrated that high mRNA expression of each PLOD gene was significantly associated with reduced OS and DFS in ccRCC patients (**Figures 3A, B**). Moreover, interaction analysis revealed that patients with both high PLOD expression and high tumor grade (Grade 3/4) had the poorest survival outcomes, whereas those with low/medium PLOD expression and low tumor grade (Grade 1/2) showed the most favorable prognosis (**Figure 3C**), underscoring the combined prognostic effect of PLOD expression and tumor grade. ROC analysis confirmed the strong diagnostic performance of all three PLOD genes, with *PLOD3* showing the highest accuracy (AUC = 0.919), followed by *PLOD1* (AUC = 0.869) and *PLOD2* (AUC = 0.838) (**Figure 3D**). Univariate Cox regression further supported their prognostic value, associating elevated expression of *PLOD1* (HR = 1.313, *p* = 0.042), *PLOD2* (HR = 1.440, *p* < 0.001), and *PLOD3* (HR = 1.402, *p* = 0.049) with increased mortality risk. Other conventional clinicopathological parameters including T stage, M stage, and tumor stage also significantly correlated with survival, consistent with clinical expectations (**Figure 3E**). However, in multivariate Cox regression adjusting for clinical covariates including age, gender, and TNM stage, the association between PLOD expression and survival was no longer significant (*PLOD1*: *p* = 0.888; *PLOD2*: *p* = 0.416; *PLOD3*: *p* = 0.736), while advanced tumor stage (HR = 2.164, *p* < 0.001) and age (*p* < 0.001) remained independent prognostic factors (**Supplementary Figure S4B**). These results indicate that while PLOD genes show strong diagnostic and univariate prognostic value, their predictive power may by mediated through established clinicopathological parameters. Nevertheless, their consistent overexpression and synergistic effect with tumor grade support their potential role as non-independent biomarkers reflecting ccRCC progression.

### Molecular landscape and tumor microenvironment interactions

Genetic alteration analysis revealed relatively low but potentially impactful mutation frequencies (0.7-3%) across PLOD family members (**Supplementary Figures S5A, B**), with significant associations with OS (**Supplementary Figure S5C**) but not DFS (**Supplementary Figure S5D**).

**Figure 4:**
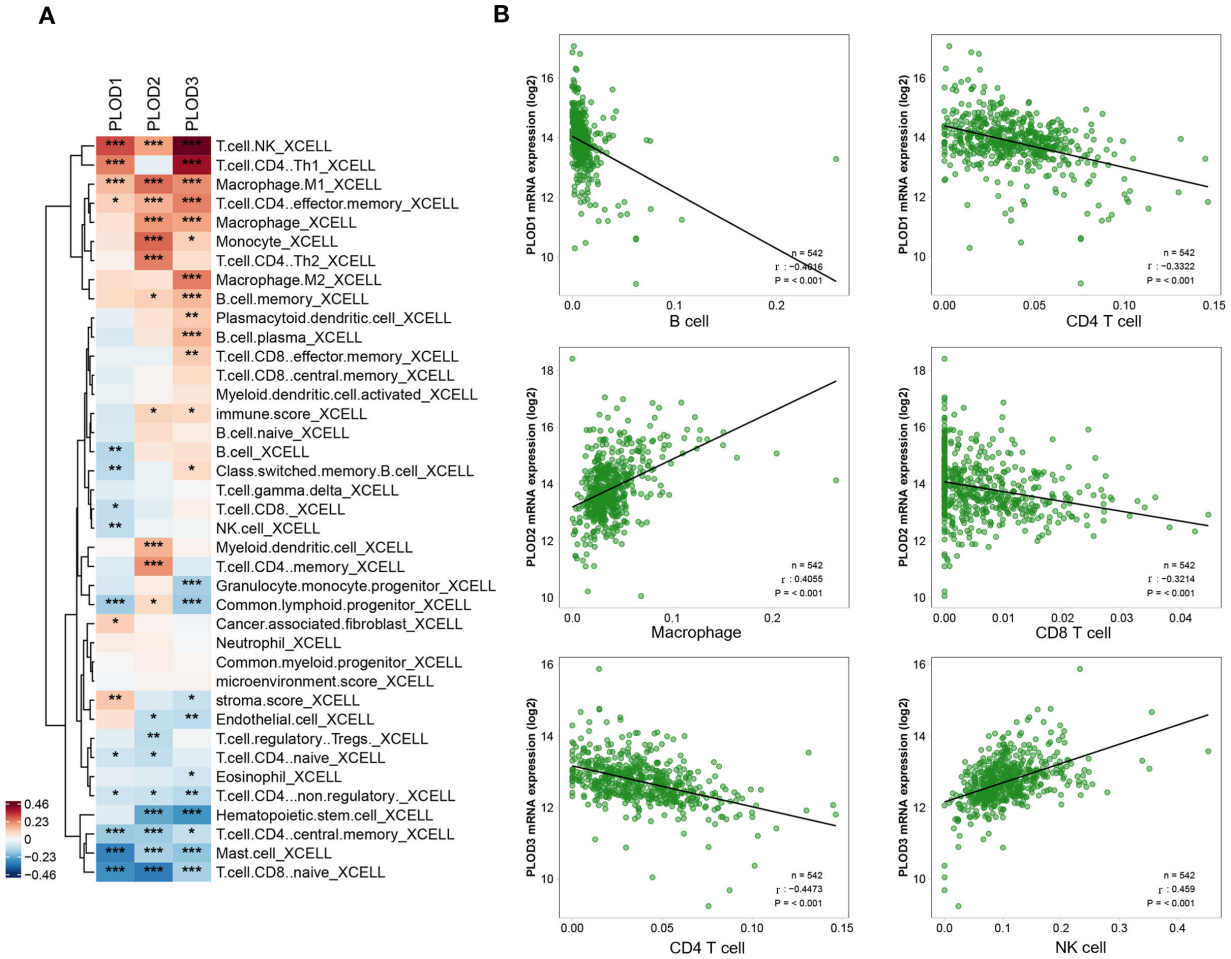
Correlations between PLOD gene expression and immune cell infiltration in ccRCC. **(A)** Heatmap showing the correlation patterns between PLOD family members and immune cell populations in ccRCC. **(B)** Spearman’s rank correlation analysis further validates the relationship between PLOD genes and key immune populations. *P* < 0.05. *P < 0.05, **P < 0.01, ***P < 0.001.

To investigate the role of PLOD gene family in the tumor immune microenvironment of ccRCC, we systematically analyzed the correlation between PLOD gene expression and immune infiltration using multiple algorithms (CIBERSORT, CIBERSORT-ABS, XCELL, EPIC, MCP-COUNTER, quanTIseq, and TIMER). As visualized by the XCELL-derived heatmap (**Figure 4A**; **Supplementary Figure S6**), distinct immune association pattern emerged for each PLOD family member with specific immune cell subsets. Notably, *PLOD1* expression correlated most strongly with cancer-associated fibroblasts, macrophage, NK cells, B cells and CD8^+^ T cells, suggesting a potential role in modulating stromal remodeling, macrophage recruitment and both innate and humoral immune response. *PLOD2* expression was closely linked to infiltration levels of macrophages, neutrophils, myeloid dendritic cells, CD8^+^ T cells, indicating a possible function in regulating myeloid immune cells activity and adaptive immunity. *PLOD3* demonstrated the broadest and robust immunomodulatory associations, showing significant correlations with macrophage, neutrophils, NK cells, B cells, CD4^+^ T cells, and CD8^+^ T cells. These findings offer new immunological insights into the mechanisms by which PLOD genes facilitate tumor progression. To further elucidate the relationship between PLOD gene expression and immune cell infiltration in the tumor microenvironment, we performed Spearman correlation analysis and selected the most significant associations for visualization. *PLOD1* expression demonstrated a significant correlation with B cells and CD8^+^ T cells, *PLOD2* expression demonstrated a significant correlation with CD8^+^ T cells and Macrophage, *PLOD3* expression showed a significant correlation with CD4^+^T cell and NK cells (**Figure 4B**). These results collectively suggest PLOD gene family are involved in distinct aspects of tumor-immune interactions.

Analysis of the GSE111360 dataset revealed distinct immune cell infiltration patterns in ccRCC, with monocyte/macrophage (Mono/Macro) cells as the predominant population, followed by NK cells (**Supplementary Figures S7A, B**). *PLOD1* and *PLOD3* expression was broadly distributed across multiple immune cell types, with particularly high expression observed in Mono/Macro cells and proliferating T cells (**Supplementary Figures S7E–G**). Consistent with these findings, analysis of the GSE139555 dataset confirmed Mono/Macro cells as the most abundant immune population, with conventional CD4^+^ T cells (CD4 Tconv) and B cells forming secondary populations (**Supplementary Figures S7C, D**). In this dataset, *PLOD1* and *PLOD3* expression showed more pronounced localization, with strong expression detected in Mono/Macro cells and dendritic cells (**Supplementary Figures S7H–J**). These consistent patterns across independent datasets suggest a potential role for PLOD-expressing immune cells, particularly Mono/Macro cells, in shaping the ccRCC tumor microenvironment.

### Regulatory network and functional associations of PLOD genes in ccRCC

**Figure 5:**
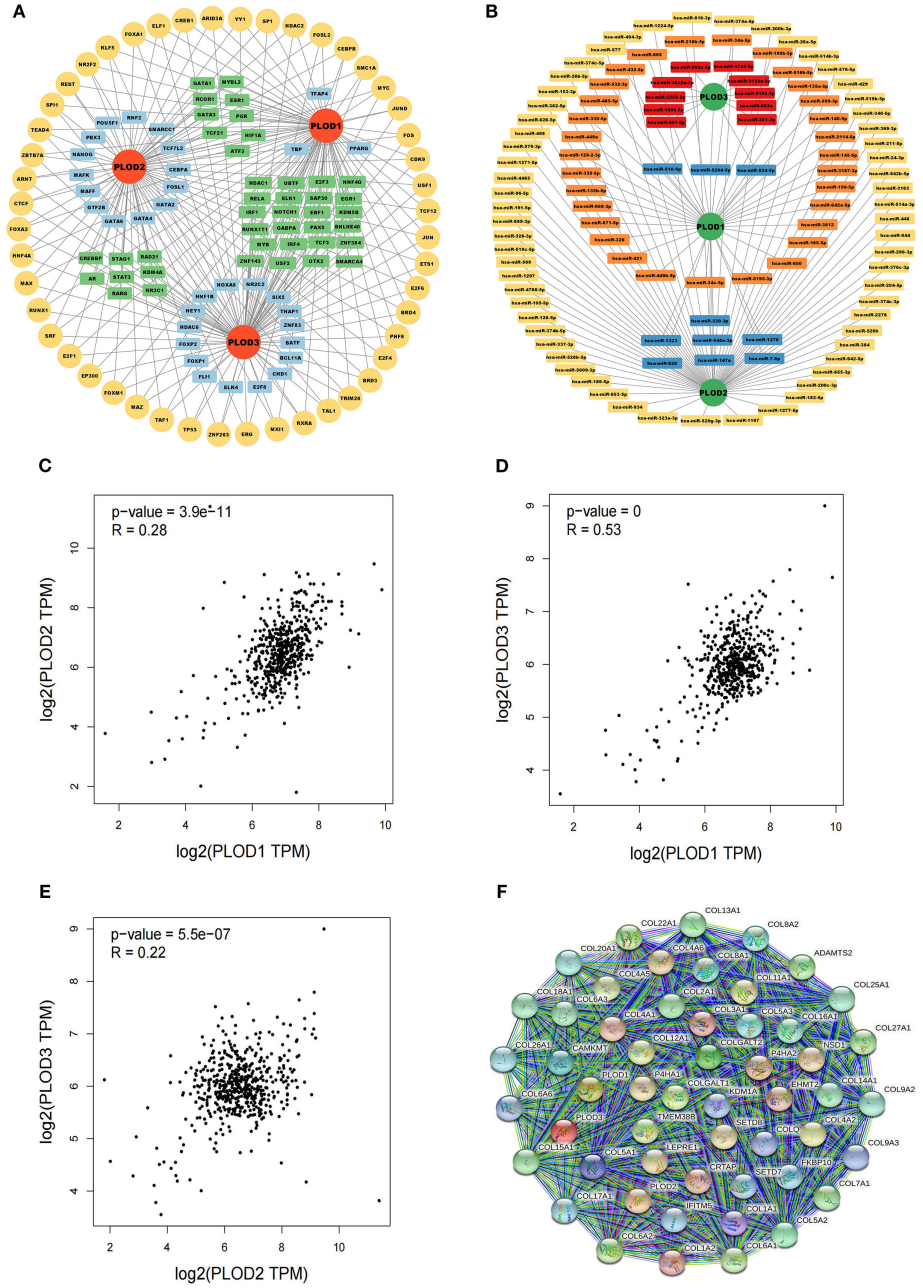
TF-targets, miRNAs-targets and co-expressed genes that regulating PLOD gene family. **(A)** TF-targets. Yellow, common TFs associated with *PLOD1*, *PLOD2* and *PLOD3*. Green, TFs associated with two PLOD genes. Blue, TFs associated with one PLOD gene. **(B)** miRNAs-targets. Orange, miRNAs associated with *PLOD1*. Yellow, miRNAs associated with *PLOD2*. Red, associated with *PLOD3*. Blue, miRNAs associated with two PLOD genes. **(C–E)** Correlations among *PLOD1*, *PLOD2* and *PLOD3* derived from GEPIA database. **(F)** Protein interaction network of 50 functional proteins with confidence score of > 0.4 based on STRING database.

MicroRNAs (miRNAs) represent a crucial class of non-coding RNAs that modulate gene expression through binding to 3’UTR regions of target mRNAs, playing pivotal roles in maintaining cellular homeostasis and responding to environmental cues (24, 25). To elucidate the regulatory landscape of PLOD genes in ccRCC, we employed an integrated bioinformatics approach combining TF-target (hTFtarget and Genecards) and miRNA-target (Starbase and Targetscan) databases. Our analysis identified 123 TFs and 113 miRNAs potentially regulating the PLOD gene family (**Figures 5A, B**). Specifically, we found 85, 79, and 96 TFs regulating *PLOD1*, *PLOD2*, and *PLOD3*, respectively, while 42, 68, and 16 miRNAs were predicted to target *PLOD1*, *PLOD2*, and *PLOD3* (**Supplementary Figures S8**, **S9**).

To understand the functional interplay among PLOD family members, we analyzed their co-expression patterns using the GEPIA database. Significant positive correlations were observed between *PLOD1* and *PLOD2* (R = 0.28, *P* < 0.05), *PLOD1* and *PLOD3* (R = 0.53, *P* < 0.05), and *PLOD2* and *PLOD3* (R = 0.22, *P* < 0.05) (**Figures 5C–E**). Furthermore, we constructed a protein-protein interaction network using the STRING database to identify potential functional partners of PLOD genes. The top 50 co-expressed genes were selected for subsequent functional analysis (**Figure 5F**; **Supplementary Table S1**), providing insights into the molecular networks associated with PLOD activity in ccRCC pathogenesis.

### Functional analysis and validation of PLOD gene family in ccRCC progression

**Figure 6:**
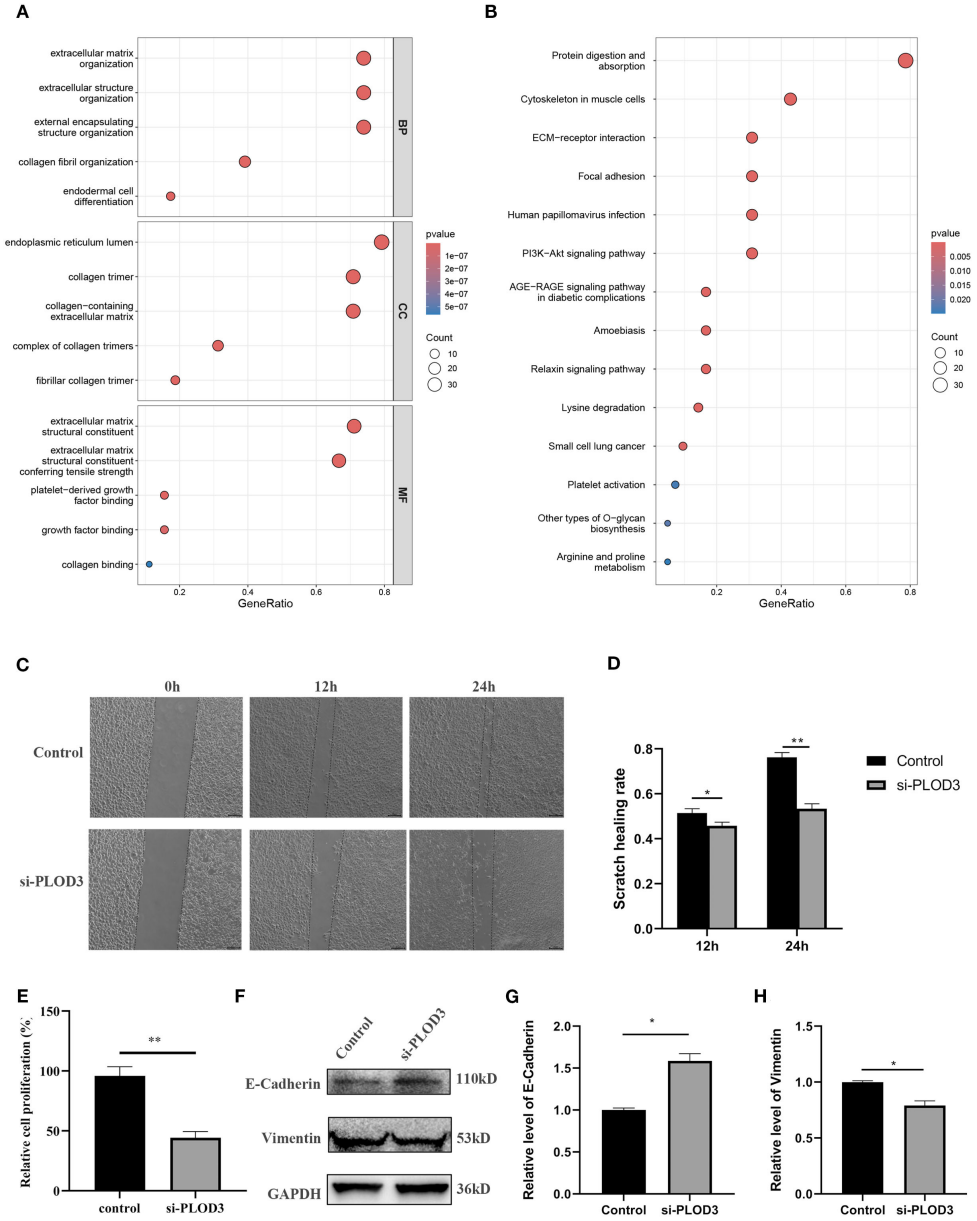
Functional analysis of PLOD gene family in ccRCC. **(A)** GO enrichment. **(B)** KEGG pathway enrichment. **(C, D)** After knocking down *PLOD3* in 786-O cells, the scratch healing rate was measured. **(E)** After knocking down *PLOD3* in 786-O cells, the proliferation rate was measured using CCK-8. **(F–H)** Western blot analysis of E-Cadherin and Vimentin in 786-O cell line with knocking down of *PLOD3*. *P < 0.05, **P < 0.01.

To elucidate the potential mechanisms through which the PLOD gene family regulates ccRCC progression, we performed comprehensive GO and KEGG pathway analyses. The GO enrichment analysis revealed significant enrichment of PLOD family members and their top 50 co-expressed genes in critical pathways including extracellular matrix organization, extracellular structure organization, collagen fibril organization (**Figure 6A**). These findings suggest that PLOD-mediated regulation of extracellular matrix dynamics plays a pivotal role in ccRCC pathogenesis. KEGG pathway analysis further demonstrated that PLOD network is primarily involved in protein digestion and absorption, ECM-receptor interaction, and lysine degradation pathways (**Figure 6B**). Notably, the PI3K-Akt signaling pathway emerged as a potentially crucial mediator of PLOD function in ccRCC progression. These enriched pathways collectively highlight the importance of extracellular matrix composition, collagen synthesis, and lysine metabolism in PLOD-mediated ccRCC development, providing valuable insights into the molecular mechanisms underlying PLOD’s oncogenic role.

Based on comprehensive analysis of clinical and experimental data, we selected the 786-O cell line, which exhibited the highest expression of *PLOD3* protein expression, for functional validation studies. Following the initial screening, the most effective siRNA was chosen to investigate the phenotypic consequences of PLOD depletion (**Supplementary Figure S10**). Wound healing assays demonstrated that PLOD3 silencing significantly impaired the migratory capacity of ccRCC cells, as shown by markedly reduced scratch wound closure rates compared to control groups (**Figures 6C, D**). Supporting these findings, CCK-8 proliferation assays revealed that PLOD3 knockdown substantially inhibited cellular proliferation in 786-O cells (**Figure 6E**). To further investigate the underlying mechanisms, we examined PLOD3’s role in epithelial-mesenchymal transition (EMT), a fundamental process in cancer metastasis. Western blot analysis showed that PLOD3 depletion led to significant upregulation of the epithelial marker E-cadherin and concurrent downregulation of the mesenchymal marker Vimentin (**Figures 6F–H**), indicating effective suppression of EMT progression. Taken together, these results establish that PLOD3 critically regulates ccRCC cell migration and progression, at least in part through modulation of EMT-related pathways.

### Drug sensitivity analysis of PLOD gene family in ccRCC

**Figure 7:**
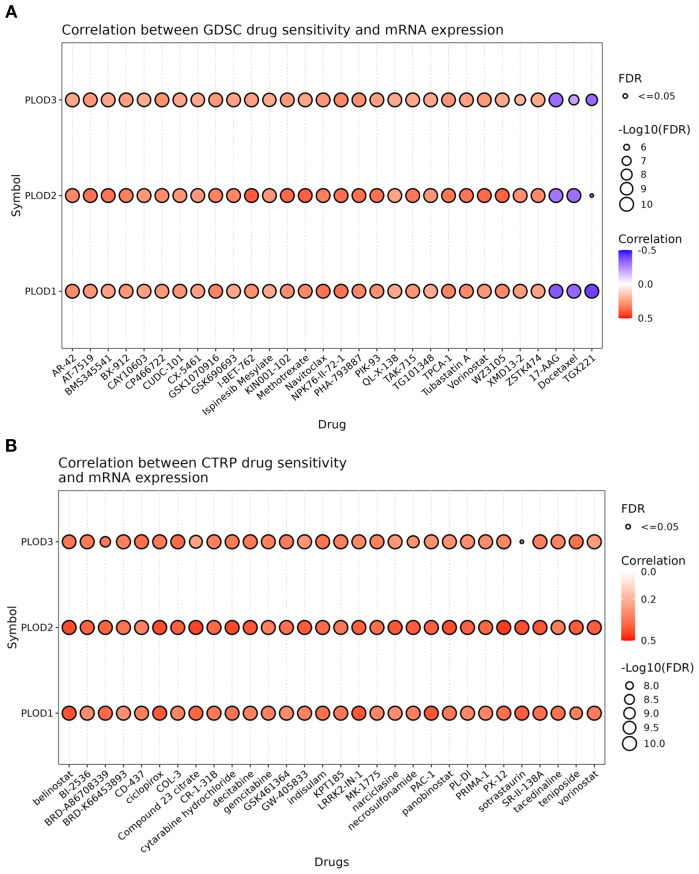
Drug sensitivity of PLOD gene family from GSCA. The bubble plot illustrates the correlations between gene expression and FDA-approved drugs. Specifically, the positive Spearman correlation coefficients suggest that elevated gene expression confers drugs resistance, as determined through GDSC **(A)** and CTRP **(B)**.

To investigate the therapeutic implications of PLOD expression, we conducted comprehensive drug sensitivity analysis using the GSCA platform integrated with GDSC and CTRP databases. Our analysis revealed a significant association between PLOD expression levels and drug response profiles. In the GDSC database, high expression of PLOD family members showed negative correlations with a limited number of therapeutic agents, while demonstrating positive correlations with the majority of tested drugs (**Figure 7A**). This pattern was further corroborated by analysis of the CTRP database, which revealed consistent positive correlations between PLOD expression and resistance to various pharmacological compounds (**Figure 7B**). These consistent findings across independent databases strongly suggest that elevated PLOD expression is associated with increased drug resistance in ccRCC. The robust correlation between PLOD expression levels and therapeutic response profiles positions the PLOD gene family as promising biomarkers for predicting drug sensitivity and guiding personalized treatment strategies in ccRCC patients.

### Prediction and molecular docking analysis of PLOD-targeting chemicals

**Figure 8:**
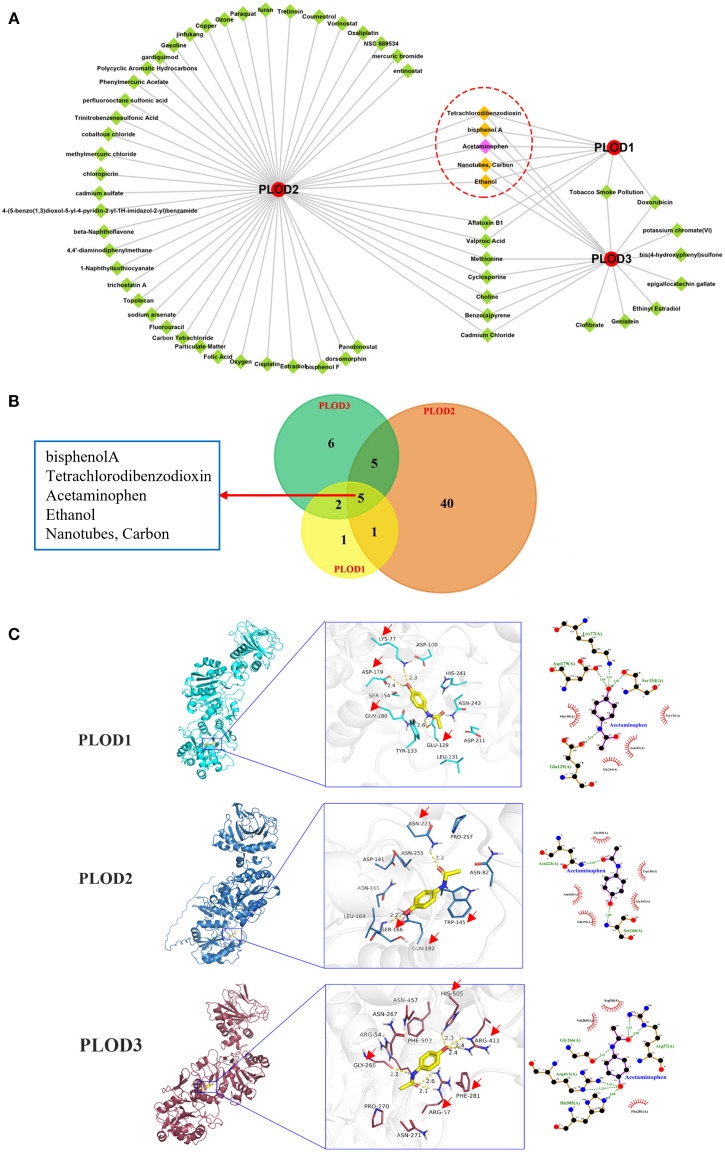
Predicted chemicals interacting with PLOD genes and the docking results. **(A)** Total 9, 51, and 18 chemicals were predicted which interacted with *PLOD1*, *PLOD2*, and *PLOD3*, respectively. Red circle means PLOD gene family, green diamond indicates chemical, yellow diamond refers to common chemical. **(B)** Venn diagram shows the numbers of chemicals that interacted with PLOD genes. **(C)** Docking results of affinity between PLOD genes and Acetaminophen. The red arrows indicate the amino acids where acetaminophen interacts with PLOD gene family.

Given the established correlation between PLOD overexpression and drug resistance, we sought to identify potential chemical modulators of PLOD activity. Using the CTD, we identified 9, 51, and 18 chemicals interacting with *PLOD1*, *PLOD2*, and *PLOD3*, respectively. Notably, five chemicals-isphenol A, tetrachlorodibenzodioxin, acetaminophen, ethanol, and nanotubes/carbon- emerged as common regulators of all three PLOD family members (**Figures 8A, B**).

To investigate the molecular interactions between these chemicals and PLOD proteins, we performed molecular docking analysis. The three-dimensional structures of *PLOD1* and *PLOD2* were obtained from UniProt, while *PLOD3* structure was retrieved from the Protein Data Bank (PDB ID: 6FXR). Acetaminophen demonstrated strong binding affinities with all three PLOD proteins, with binding energies of −6.1 kcal·mol^-1^ (*PLOD1*), −6.2 kcal·mol^-1^ (*PLOD2*), and −6.3 kcal·mol^−1^ (*PLOD3*). Detailed analysis of ligand-receptor interactions revealed that acetaminophen forms multiple hydrogen bonds and hydrophobic interactions with key residues in each PLOD protein. Specifically, in *PLOD1*, acetaminophen established hydrogen bonds with LYS77, ASP179 and GLU129, along with hydrophobic interactions with TYR223 and GLN180. For *PLOD2*, hydrogen bonds were formed with ASN223 and SER166, while hydrophobic interactions occurred with TRP145 and GLN192. In *PLOD3*, acetaminophen interacted through hydrogen bonds with ARG411, ARG57, GLY266 and HIS505, and hydrophobic interactions with PHE281 (**Figure 8C**). The extensive molecular interactions demonstrating stable binding of acetaminophen within PLOD proteins’ active sites, coupled with our comprehensive findings, establish PLOD genes as crucial players in ccRCC pathogenesis with significant clinical potential - serving not only as diagnostic and prognostic biomarkers but also revealing acetaminophen as a promising PLOD modulator for therapeutic intervention.

## Discussion

The present study provides a comprehensive analysis of the PLOD gene family in ccRCC, revealing their significant roles in tumor progression, clinical correlation, immune modulation, and potential as therapeutic targets. Our findings demonstrate that *PLOD1*, *PLOD2*, and *PLOD3* are consistently upregulated in ccRCC tissues and correlate with poor clinical outcomes, including reduced OS and DFS. Functional enrichment analysis further highlights their involvement in critical biological processes such as ECM remodeling, collagen metabolism, and lysine degradation, which are essential for tumor growth and metastasis. These results confirmed and substantially extended the findings of Xu et al. (18). We validated the prognostic significance of PLOD overexpression in ccRCC and provided novel evidence linking PLOD expression to immune infiltration patterns, suggesting a potential role in shaping the tumor microenvironment. Furthermore, systematic drug sensitivity analysis associated PLOD expression with resistance to multiple therapeutic agents, implicating that PLODs not only as biomarkers but also as functional contributors to treatment resistance. Finally, we identified acetaminophen as a potential PLOD-binding compound. Thus, while Xu et al. established a prognostic framework, our work advances the understanding of PLODs as active regulators of tumor immunity and treatment response, highlighting their potential as therapeutic targets in ccRCC.

The oncogenic roles of PLOD genes are not unique to ccRCC but are conserved across multiple malignancies. *PLOD1* overexpression has been reported in gastric cancer, glioma, and osteosarcoma, where it promotes tumor growth and metastasis (13, 26–30). *PLOD2*, a key enzyme in collagen crosslinking, is associated with poor outcomes in oral squamous cell carcinoma, hepatocellular carcinoma, and breast cancer (31–35). Similarly, *PLOD3* is overexpressed in ovarian cancer, colon adenocarcinoma, and hepatocellular carcinoma, where it facilitates tumor progression (36–38). These studies collectively highlight the role of PLOD genes in ECM remodeling and tumor microenvironment modulation, suggesting that targeting PLOD-mediated pathways may have broad therapeutic implications across cancer types.

Functional enrichment analysis demonstrates that the PLOD gene family plays a central role in collagen cross-linking and ECM-receptor interactions. *PLOD1* enhances stable cross-link formation and matrix stiffness, *PLOD2* regulates fibrillar collagen alignment and lysine hydroxylation essential for mature cross-links, and *PLOD3* facilitates collagen glycosylation and basement membrane organization. Their collective upregulation promotes ECM restructuring, increasing tissue stiffness and collagen alignment, and activating mechanosensitive signaling via integrins (11). For instance, hypoxia upregulates *PLOD2* expression via HIF1A, and *PLOD2* promotes the malignant progression of clear cell renal cell carcinoma by binding to and activating the EGFR/AKT signaling pathway, while the *PLOD2* inhibitor minoxidil suppresses this process, suggesting *PLOD2* as a potential prognostic marker and therapeutic target for ccRCC (39). Similarly, *PLOD3* knockdown suppressed ccRCC malignancy by downregulating TWIST1 expression, thereby inhibiting β-catenin and AKT signaling pathways (40). These finding collectively suggest that while *PLOD2* and *PLOD3* operate through distinct upstream regulators, they converge on common oncogenic pathways including AKT activation.

Although this study identifies significant correlations between PLOD gene expression and immune cell infiltration through bioinformatic analyses, the mechanistic insights into how individual PLOD family members shape the tumor immune microenvironment remain incompletely understood. Previous evidence suggests that *PLOD2*-driven collagen cross-linking enhances ECM stiffness and promotes dense, aligned fiber architectures that physically impede the infiltration of cytotoxic T cells and NK cells (39). Similarly, *PLOD1* contributes to stable collagen cross-links and stromal fibrosis, likely impairing T-cell mobility, while *PLOD3* may influence immune cell transmigration and spatial distribution through its regulation of basement membrane organization and collagen glycosylation (11, 41). These mechanisms position PLOD-mediated ECM remodeling as a promising therapeutic target to reverse immune suppression and improve response to immunotherapies in ccRCC.

Interestingly, our bioinformatic analysis also revealed a positive correlation between *PLOD3* expression and NK/T cell infiltration, suggesting a potential role in establishing an immune-permissive niche—possibly via *PLOD3*-driven ECM remodeling and collagen cross-linking, which may promote immune cell migration as indicated in previous studies (18, 42). However, as this association is derived solely from computational analyses without experimental validation, we emphasize the preliminary nature of this finding. We explicitly acknowledge that the mechanistic basis of *PLOD3*-immune crosstalk remains speculative and merits further investigation through functional assays such as co-culture systems and NK cytotoxicity experiments.

Furthermore, single-cell RNA sequencing analysis uncovered substantial heterogeneity in PLOD expression across different cell types within ccRCC tumor microenvironment. This heterogeneity is observed not only between malignant cells and stromal components—such as cancer-associated fibroblasts and endothelial cells—but also among distinct malignant subclones. Such variation underscores the significant role of PLOD-driven collagen modification and ECM remodeling in promoting regional fibrosis and shaping functional tumor niches, with direct implications for therapeutic targeting. For example, tumor subpopulations with high PLOD expression often localize to fibrotic and stiffened areas that may impede the infiltration of drugs and immune cells. Therefore, therapeutic strategies targeting PLOD activity or downstream ECM cross-linking could normalize matrix architecture, enhance drug penetration, and mitigate microenvironment-mediated treatment resistance.

In addition to fostering an immunosuppressive microenvironment, PLOD-driven ECM fibrosis and increased stromal stiffness creates a physical barrier that limits drug penetration, reducing effective chemotherapeutic concentration at target sites. Concurrent activation of integrin-mediated signaling pathways, such as EGFR/AKT, which promote cell survival and diminish apoptosis in response to treatment. Thus, the PLOD family facilitates a multifunctional mechanism of resistance that combines biophysical barrier formation with pro-survival signaling activation. These insights underscore the potential of targeting PLOD-mediated ECM remodeling as a strategy for re-sensitizing ccRCC to conventional therapeutics.

This study thoroughly evaluated the prognostic value of the PLOD family using a multivariate Cox regression model. Although univariate analysis indicated that high expression of PLODs was significantly associated with poor prognosis, this correlation disappeared after adjusting for key clinical variables, including tumor stage. This suggests that the expression of PLOD gene family may not serve as an independent prognostic predictor but is closely related to tumor progression and advanced clinicopathological characteristics. Our findings link the function of PLOD genes to malignant tumor progression. It is speculated that PLOD gene family may drive tumor invasion and metastasis by promoting collagen cross-linking and tumor microenvironment stiffening, thereby leading to more advanced T stage and tumor stage, which ultimately affect patient survival. Thus, PLOD gene family may function more as biomarkers of “tumor aggressiveness” rather than direct prognostic predictors.

While our study provides comprehensive analysis in the role of the PLOD gene family in ccRCC progression, several limitations should be acknowledged. First, the limited sample size of patient tissues and the use of only two ccRCC cell lines may not adequately capture the considerable heterogeneity of this disease. Future studies should validate these findings in larger, multi-institutional patient cohorts and a broader array of cellular models to strengthen the mechanistic relevance of our results. Second, our study focused on knockdown experiments to validate the effects of the PLOD gene family on tumor metastasis. However, combining both knockdown and overexpression experiments would provide a more comprehensive validation of our findings. Third, we did not perform *in vivo* experiments, which could better simulate the real conditions of human tumor tissues. Furthermore, we did not explore the molecular mechanisms underlying PLOD-mediated regulation of ccRCC invasion and metastasis in depth. For instance, the involvement of key signaling pathways (e.g., PI3K/AKT, HIF-1α, or TGF-β) and downstream effectors remains to be elucidated. Finally, while molecular docking analysis identified acetaminophen as a potential PLOD inhibitor, it is important to emphasize that this finding remains preliminary. The docking results solely represent computational predictions. Rigorous validation through both *in vitro* and *in vivo* experiments is essential to confirm the interaction between acetaminophen and PLOD proteins and to evaluate its therapeutic efficacy in ccRCC. These steps are critical to translating this preliminary finding into a viable therapeutic strategy. All above issues warrant future studies.

Our study reveals that PLOD family expression is closely associated with ECM remodeling and immunosuppression in ccRCC, suggesting potential synergistic mechanisms with existing therapeutic targets. For instance, PLOD-mediated collagen cross-linking and fibrosis may limit the efficacy of drug penetration and immune cells infiltration. Therefore, combining PLOD inhibition with VEGF-targeted agents or immunotherapies could normalize the tumor stroma, and promote an immune-permissive microenvironment. Furthermore, we will investigate the relationship between PLOD expression and established molecular subtypes of ccRCC. Differences in PLOD expression across subtypes may reflect distinct stromal activation patterns and ECM organization, which could inform subtype-specific therapeutic strategies targeting the tumor microenvironment.

Elevated PLOD expression shows promise as a prognostic biomarker in ccRCC, adding complementary value to existing models like IMDC or SSIGN by capturing ECM remodeling and fibrosis levels not reflected in current clinical parameters. However, clinical translation requires standardized assays for PLOD detection in routine samples, validation in large multi-institutional cohorts to confirm its additive prognostic role, and strategies to overcome tumor heterogeneity-related sampling bias. Therapeutically, inhibiting PLOD-mediated ECM cross-linking could normalize stroma, improve drug delivery, and overcome resistance, potentially in combination with anti-angiogenics or immunotherapies.

## Conclusion

In conclusion, our study establishes the PLOD gene family as critical regulators of ccRCC progression, with significant implications for prognosis and therapeutic targeting. The upregulation of *PLOD1*, *PLOD2*, and *PLOD3* in ccRCC tissues, their association with poor clinical outcomes, and their roles in ECM remodeling and immune modulation highlight their potential as diagnostic biomarkers and therapeutic targets. While our findings provide valuable insights into the molecular mechanisms underlying ccRCC progression, further research is needed to fully elucidate the functional roles of PLOD genes and translate these discoveries into clinical applications. The conserved nature of PLOD-mediated ECM modifications across cancers suggests that our findings may have broader relevance, offering new directions for cancer research and therapy development.

## Data Availability

The original contributions presented in the study are included in the article/**Supplementary Material**. Further inquiries can be directed to the corresponding authors.
