# Supplementary material for: Prognostic biomarker and clinical significance of PLOD gene family in clear cell renal cell carcinoma
Source: Front Oncol. 2025 Oct 10;15:1613540. doi: 10.3389/fonc.2025.1613540 (PMC12549286; doi:10.3389/fonc.2025.1613540)
Supplement: Supplementary file 1 [file Table1.docx]

**Prognostic biomarker and clinical significance of PLOD gene family in clear cell renal cell carcinoma**

Xuan Shang^1,2,3,†^, Liu Liu^1,2,3,†^, Zhenwei Yang^1,2^, Min Yan^1,2^, Ruimin Ren^4^, Kexin Guo^1,2^, Jie Wang^1,2^, Wei Zhang^5^, Jiasong Chang^1,2,3^, Jialei Li^1,2^, Jimin Cao^1,2,3^, Guang Li^6,*^, Lijuan Gao^1,2,3,*^,

^1^ Department of Cardiology, The First Hospital and First College of Clinical Medicine, Shanxi Medical University, Taiyuan, 030001, China

^2^ Key Laboratory of Cellular Physiology at Shanxi Medical University, Ministry of Education, Taiyuan, 030001, China

^3^ Department of Physiology, Shanxi Medical University, Taiyuan, 030001, China

^4^ Department of Urology, Shanxi Bethune Hospital (Third Hospital of Shanxi Medical University), Taiyuan, 030001, China

^5^ Department of Urology, The First Hospital and First College of Clinical Medicine, Shanxi Medical University, Taiyuan, 030001, China

^6^ Department of Cardiology, The Aﬃliated Hospital of Southwest Medical University and Key Laboratory of Medical Electrophysiology, Ministry of Education & Institute of Cardiovascular Research, Southwest Medical University, Luzhou, 646000, Sichuan, China

^†^ These authors contributed equally to this work.

^*^ Correspondence:

Lijuan Gao, MS. Email: gaolijuan@sxmu.edu.cn. ORCID: 0000-0002-2274-8445

Guang Li, PhD. Email: liguang@swmu.edu.cn.

**1. Supplementary Figures**


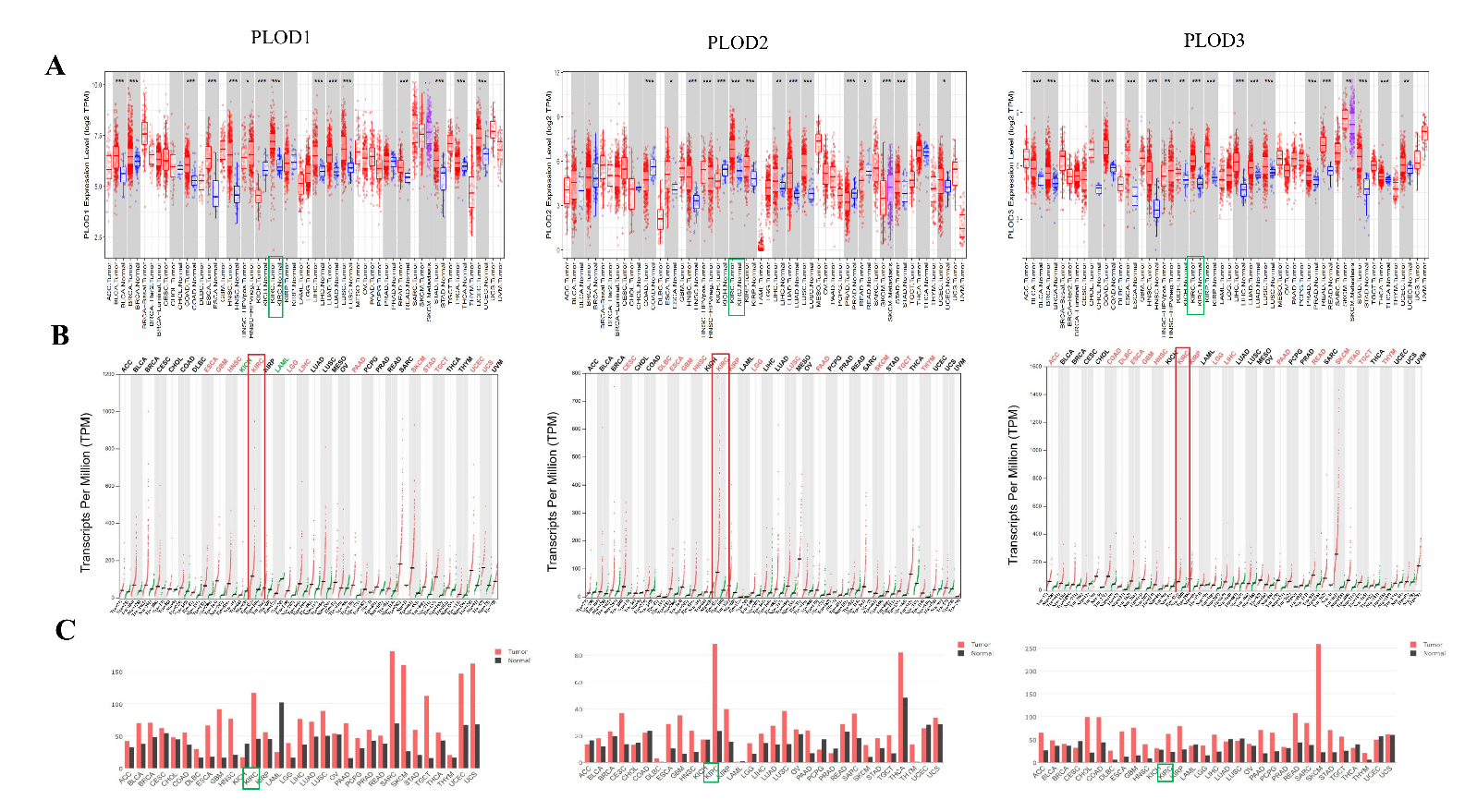


**Figure S1.** The mRNA expression levels of PLOD gene family in different types of human cancer compared with normal tissues. Note that *PLOD1*, *PLOD2* and *PLOD3* were highly expressed in KIRC. **(A)** Results of TIMER dataset showing the transcription levels of *PLOD1*, *PLOD2* and *PLOD3* in different types of cancer tissues and matched normal tissues. **(B-C)** Results of GEPIA database analysis. Potplot and boxplot showed the mRNA expression of *PLOD1*, *PLOD2* and *PLOD3* in all kind of cancers. * *P* < 0.05, *** *P* < 0.001. KIRC, kidney renal clear cell carcinoma (an alternative name of ccRCC).

**
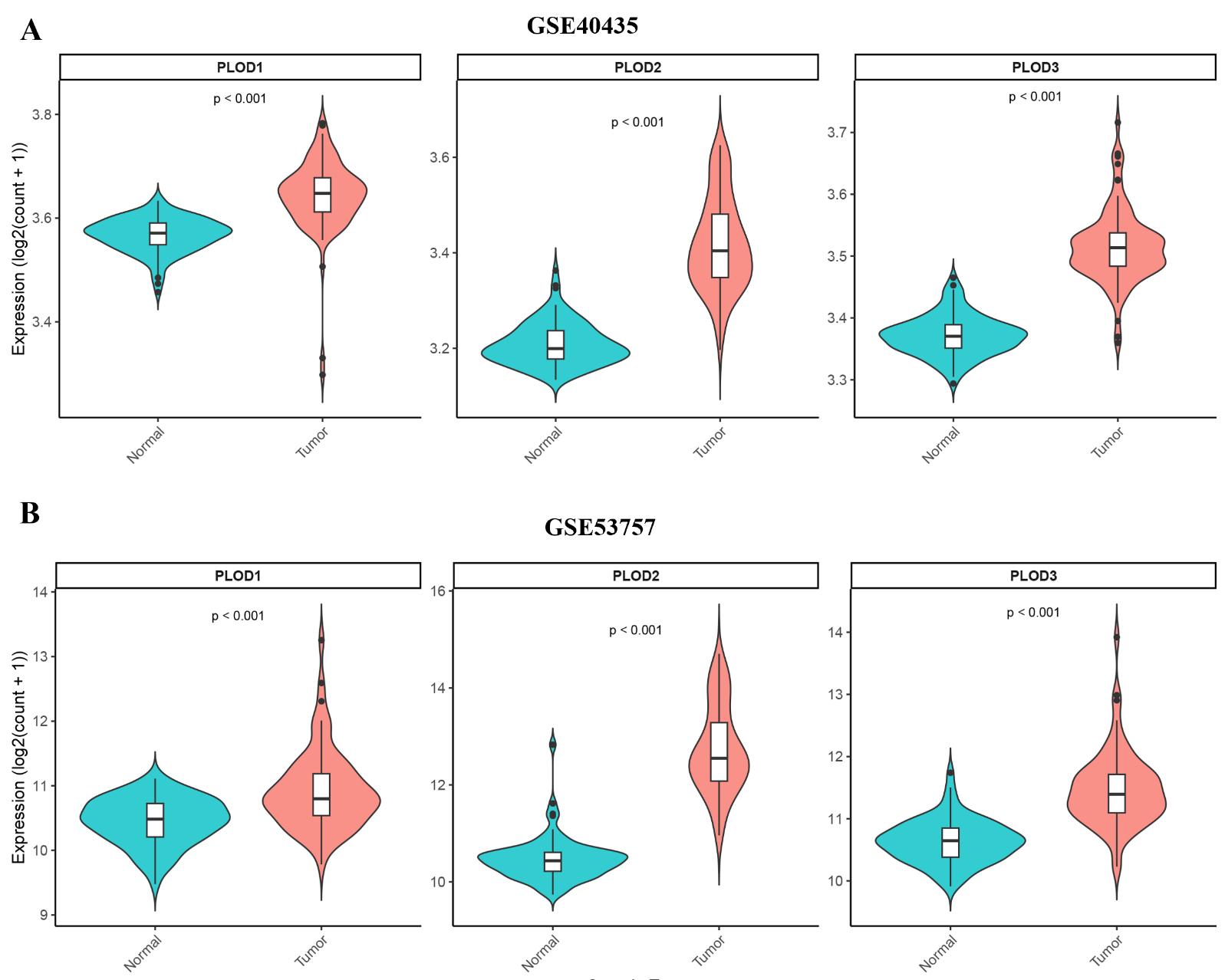
**

**Figure S2.** Differential expression analysis of PLOD gene family in GEO datasets. **(A)** PLOD gene family upregulated in ccRCC patients compared with normal patients in GSE40435 datasets. **(B)** PLOD gene family upregulated in ccRCC patients compared with normal patients in GSE53757 datasets.


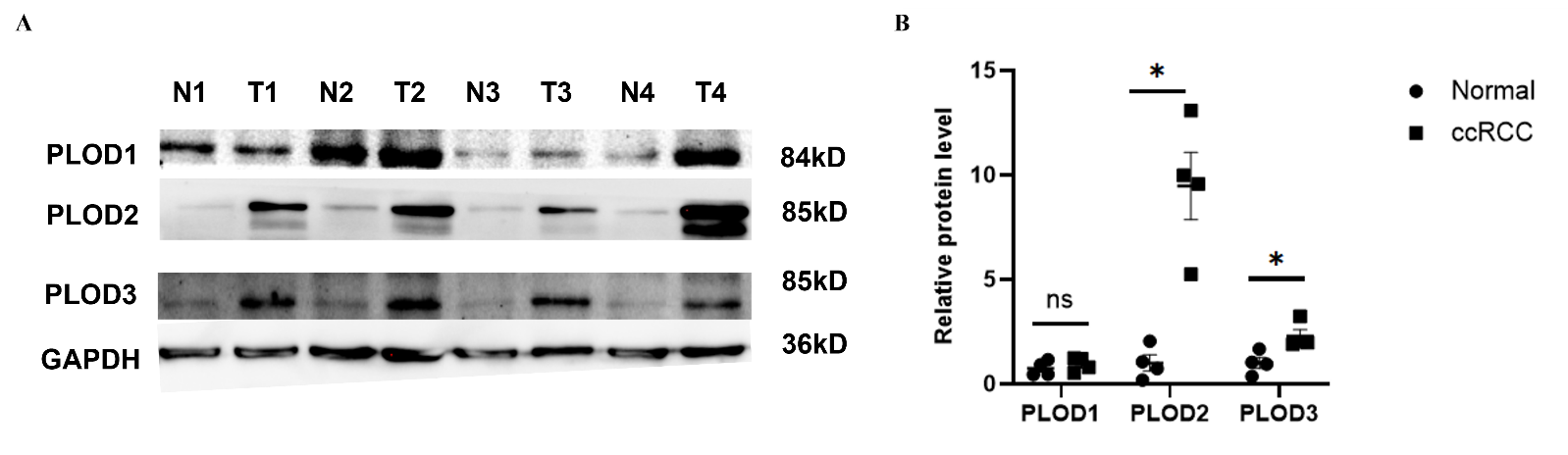


**Figure S3.** Protein expression analysis of PLOD gene family in ccRCC patients. **(A)** Representative western blot images showing expression levels of PLOD gene family in ccRCC tissues (T) and adjacent normal kidney tissues (N) (n=4). **(B)** Quantitative analysis of PLOD protein expression levels from (A), as determined by ImageJ software.


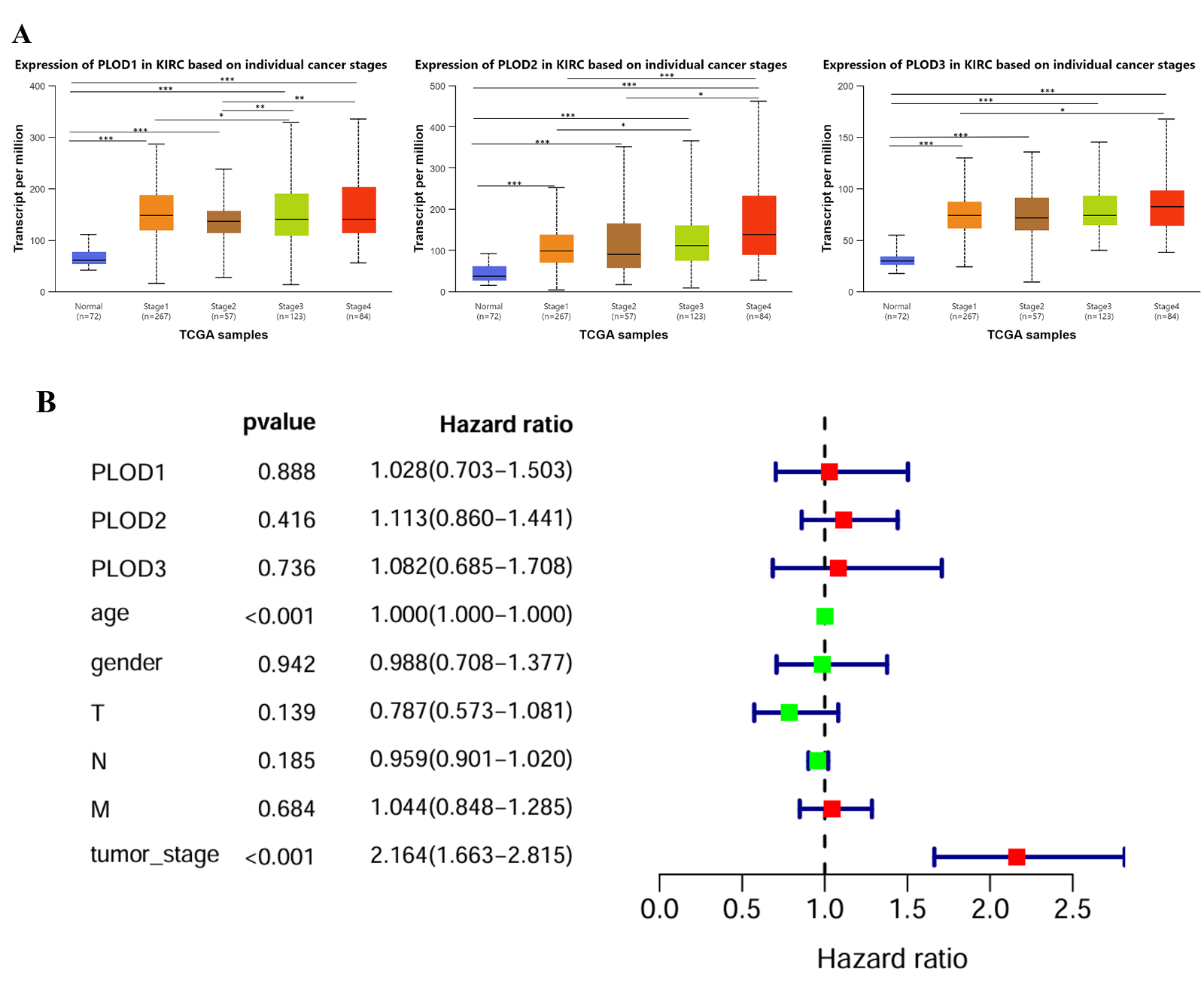
**Figure S4.** Prognostic significance of PLOD gene family in ccRCC. **(A)** Boxplot show the relative expressions of *PLOD1*, *PLOD2*, and *PLOD3* in normal individuals and ccRCC patients with different clinical stages using UACLAN. *** *P* < 0.001. **(B)** Forest plot of multivariate Cox regression analysis for PLOD genes and clinicopathological parameters, showing HR with 95% confidence intervals.


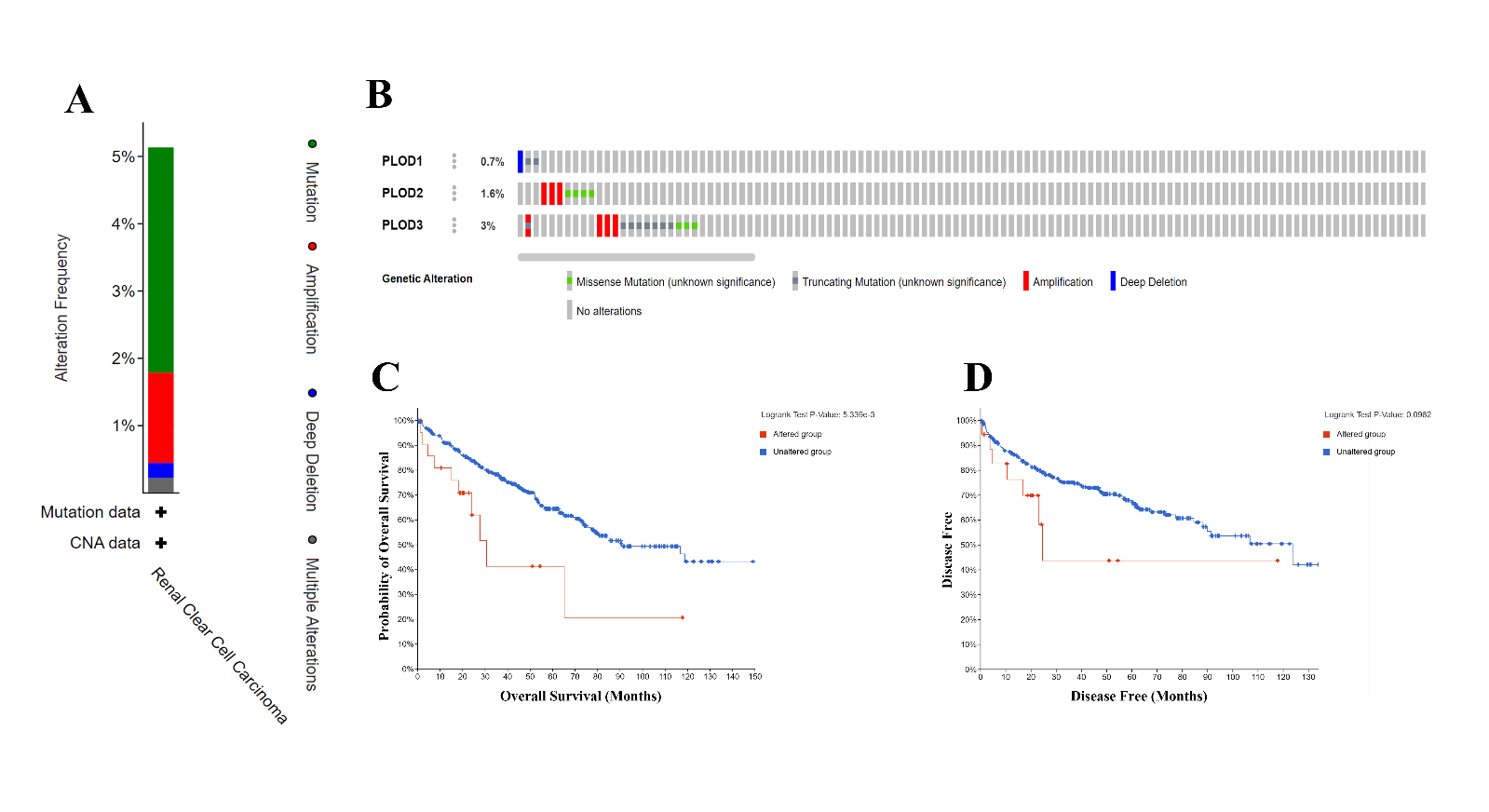


**Figure S5.** Genetic alteration frequencies of PLOD genes and their associations with survival of ccRCC using cBioPortal. (**A**) Alteration frequency of PLOD genes according to cBioPortal database. (**B**) The genetic alterations of PLOD genes in ccRCC. (**C**) and (**D**) OS and DFS of ccRCC patients with altered (red) and unaltered (blue) mRNA expression of PLOD gene family.


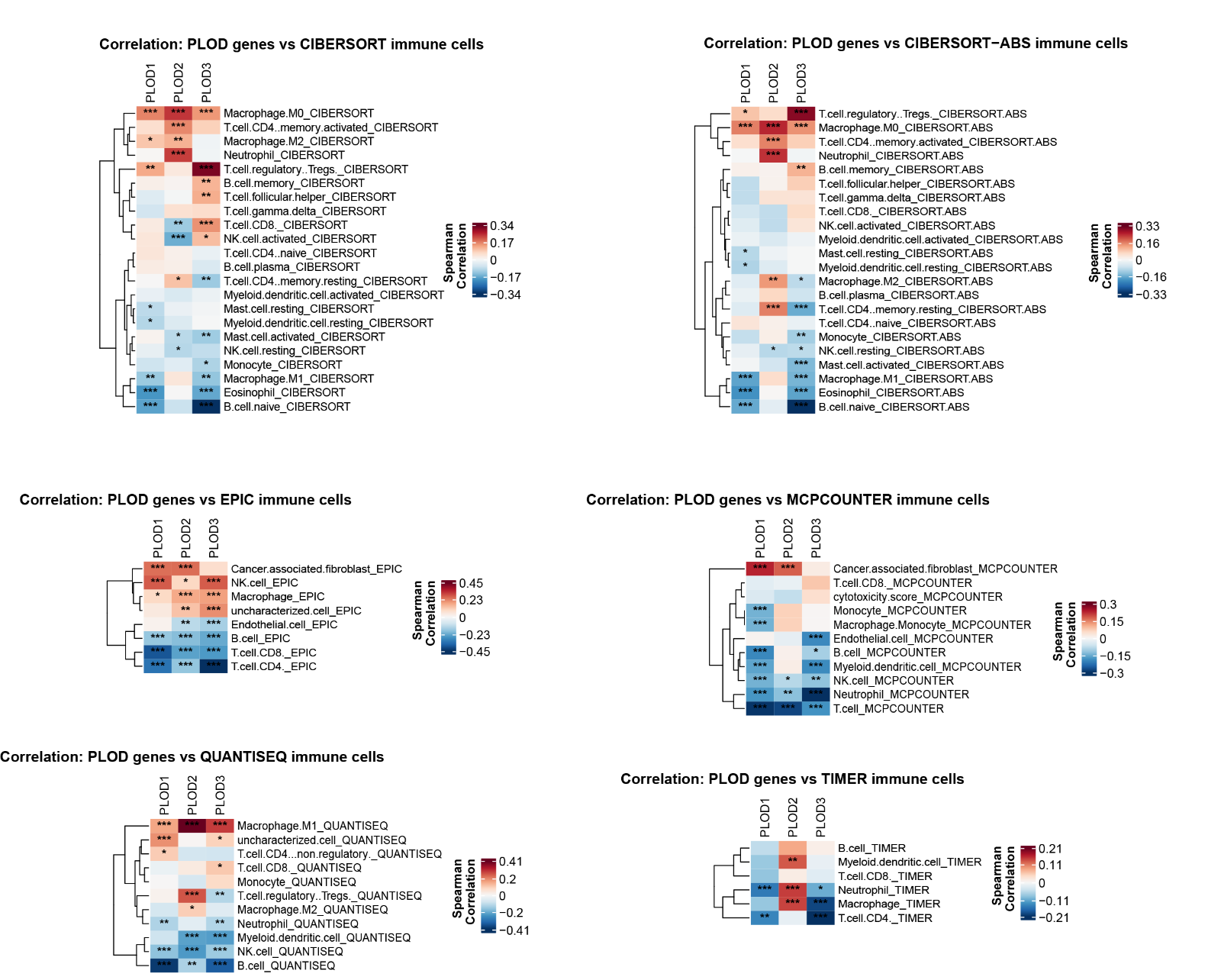


**Figure S6.** Correlation analysis between PLOD gene expression and immune cell infiltration levels in ccRCC based on multiple algorithms. Heatmaps depict the correlation coefficients of PLOD1, PLOD2, and PLOD3 expression with abundances of various immune cell types, as estimated by CIBERSORT, CIBERSORT-ABS, EPIC, MCPCOUNTER, QUANTISEQ and TIMER algorithms. Each row represents an immune cell subtype, and each column represents a PLOD gene. Red indicates positive correlation, blue indicates negative correlation, and the color intensity reflects the strength of the correlation.


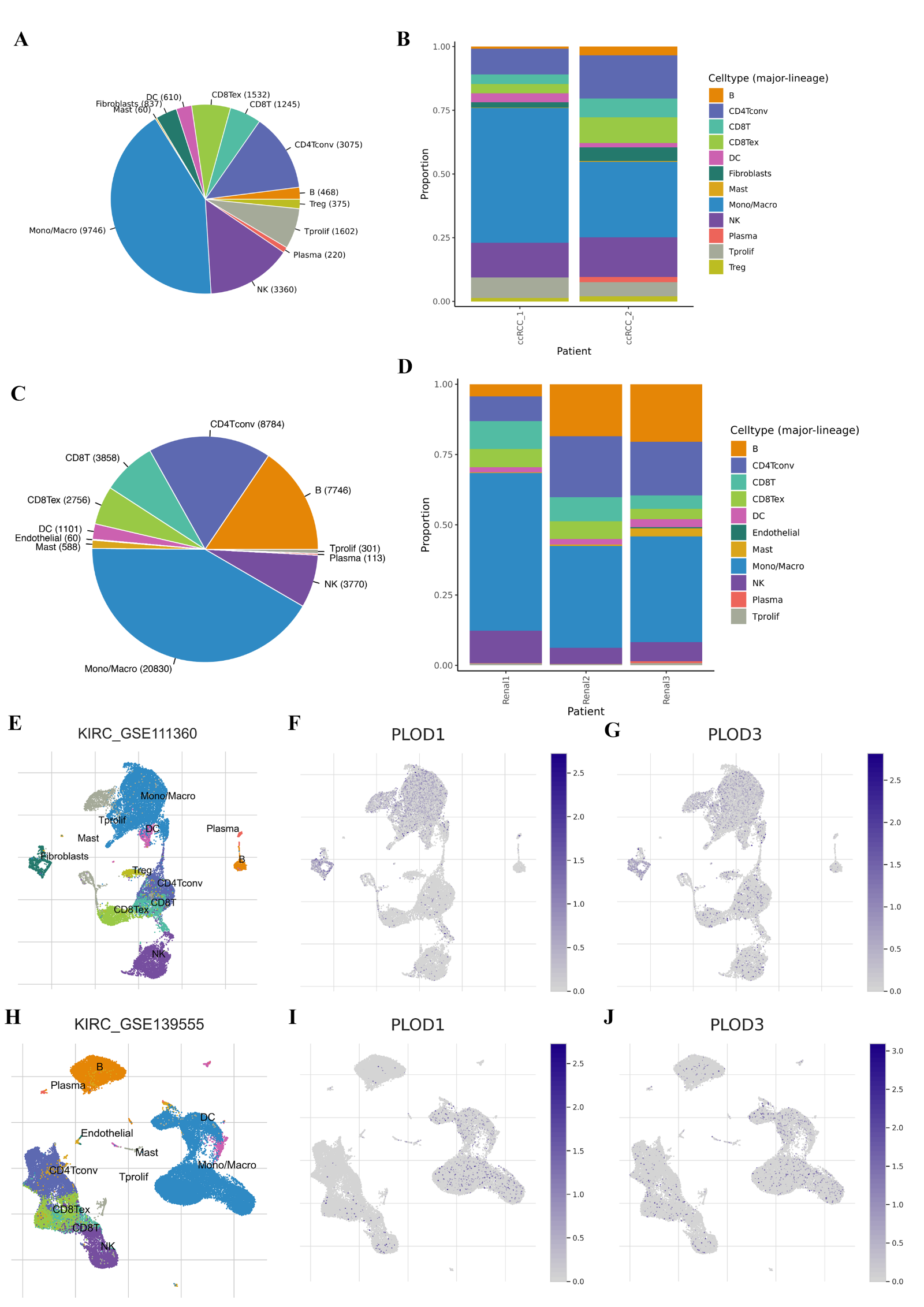


**Figure S7.** Percentage of KIRC_GSE111360 (**A**) cell numbers and KIRC_GSE139555 (**C**). Cell type distribution of KIRC_GSE111360 (**B**) and KIRC_GSE139555 (**D**). Distribution of cells expressing *PLOD1* and *PLOD3* in KIRC_GSE111360 (**E**) and KIRC_GSE139555 (**H**). Distribution of *PLOD1* in different cells in KIRC_GSE111360 (**F**) and KIRC_GSE139555 (**I**). Distribution of *PLOD3* in different cells in KIRC_GSE111360 (**G**) and KIRC_GSE139555 (**J**).


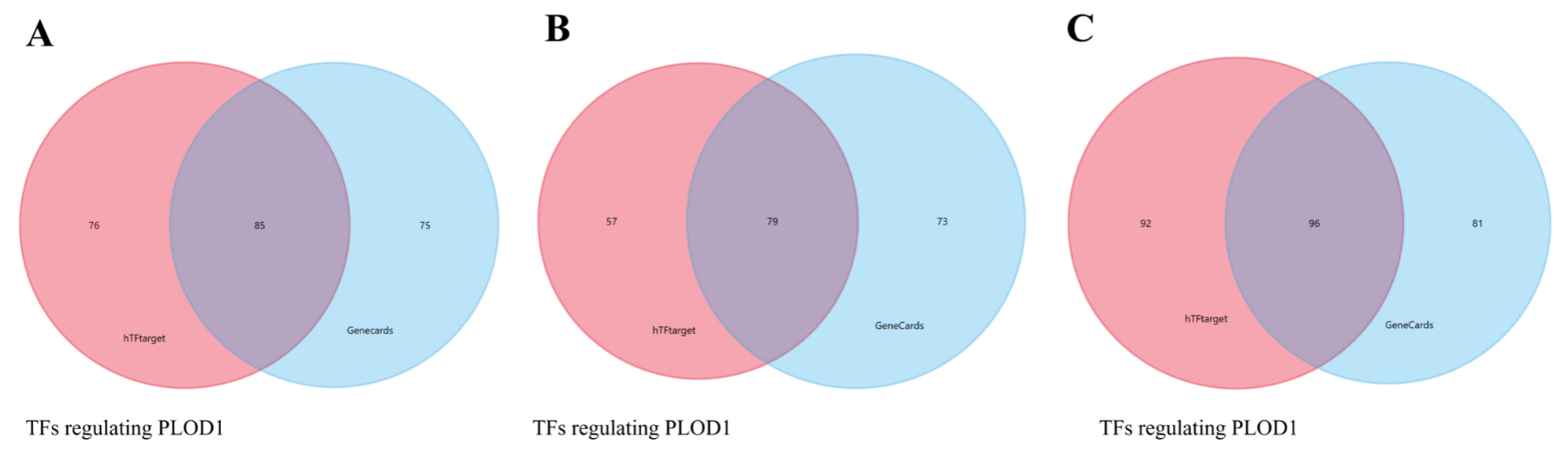


**Figure S8**. The co-expressed TFs that regulate the expressions of *PLOD1*, *PLOD2*, *PLOD3*. Data were mined from GeneCards and hTFtarget databases.

**Figure S9.** The co-expressed miRNAs that regulate the expressions of *PLOD1*, *PLOD2*, *PLOD3*. Data were mined from Starbase and Targetscan databases.
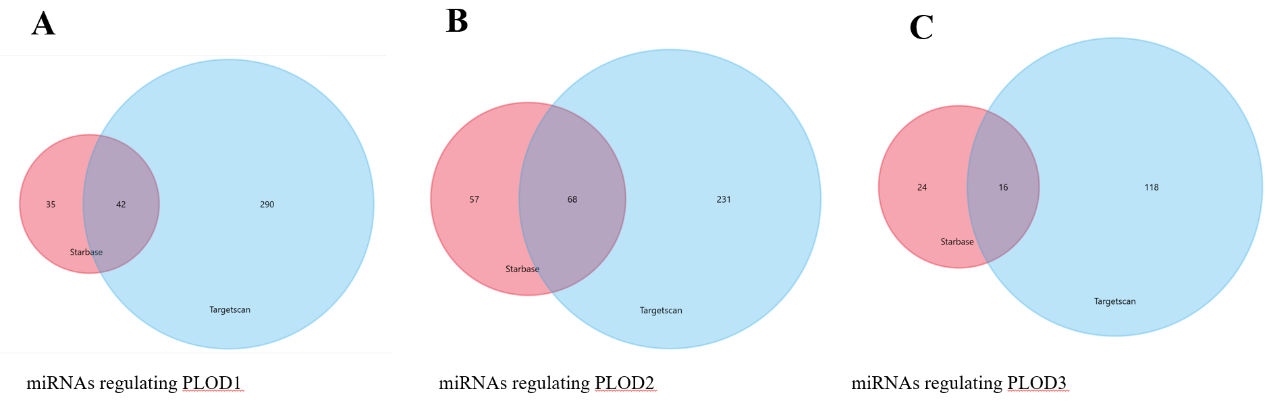


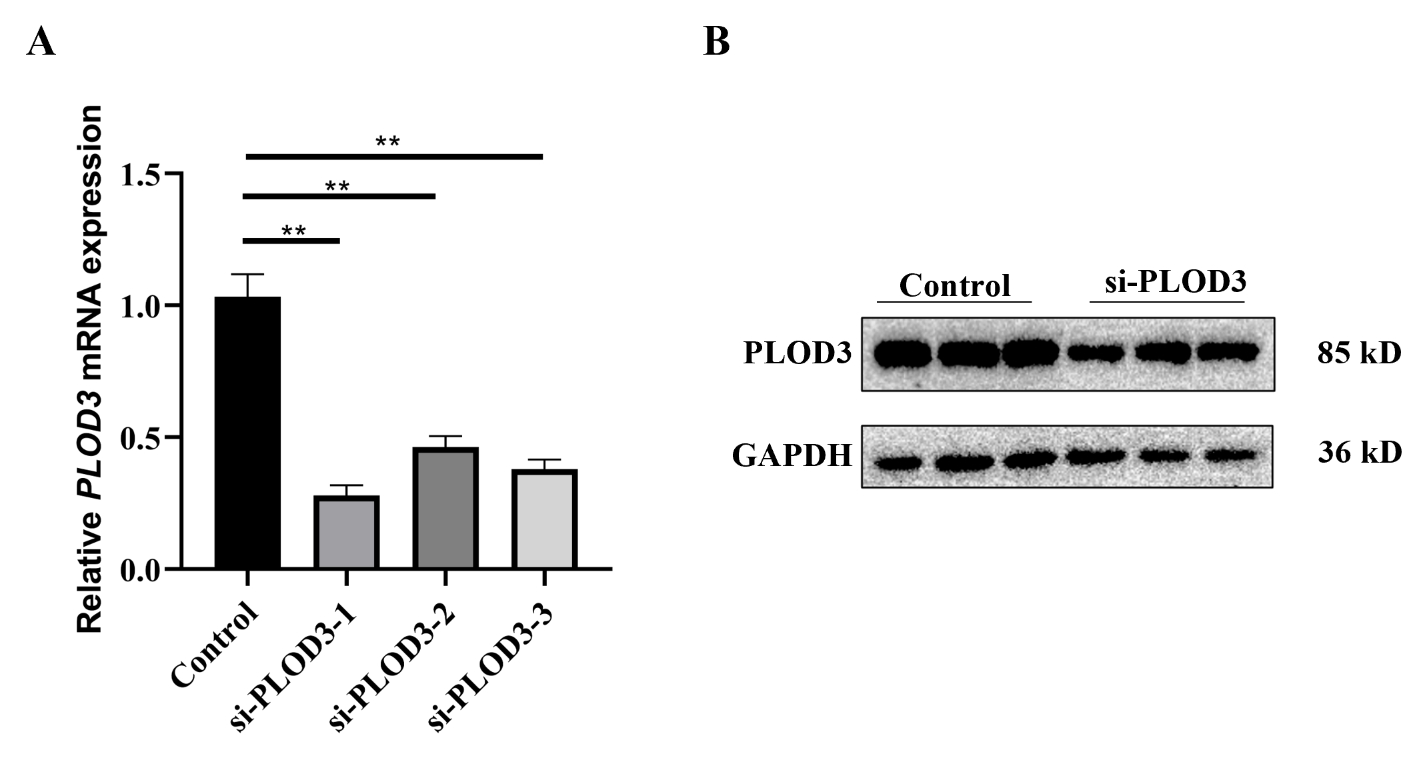


**Figure S10.** Efficient knockdown of PLOD3 by siRNA at both mRNA and protein levels in 786-O cells. (**A**) After transfecting 786-O cells with three different siRNA for 48 hours, qPCR was used to examine PLOD3 expression at the mRNA level. The results indicated that all three siRNAs effectively suppressed PLOD3 expression. (**B**) Verification the inhibitory effect of si-PLOD3-1 at the protein level using western blot analysis, demonstrating a significant decrease in PLOD3 protein expression.

**2. Supplementary Tables**

**Table S1.** The top 50 genes most relevant to the PLOD gene family

| Predicted Functional Partners | Score | Predicted Functional Partners | Score | Predicted Functional Partners | Score | Predicted Functional Partners | Score | Predicted Functional Partners | Score |
| --- | --- | --- | --- | --- | --- | --- | --- | --- | --- |
| COL5A2 | 0.982 | P4HA2 | 0.92 | COLGALT2 | 0.671 | COL13A1 | 0.539 | COL4A1 | 0.487 |
| COL1A2 | 0.977 | P4HA1 | 0.909 | COL4A2 | 0.665 | COLQ | 0.536 | COL6A3 | 0.478 |
| COL5A1 | 0.972 | EHMT2 | 0.867 | COLGALT1 | 0.627 | COL25A1 | 0.53 | COL15A1 | 0.473 |
| COL1A1 | 0.97 | CAMKMT | 0.867 | IFITM5 | 0.607 | COL4A6 | 0.53 | COL12A1 | 0.472 |
| COL3A1 | 0.967 | SETD7 | 0.854 | COL7A1 | 0.606 | COL22A1 | 0.525 | COL6A6 | 0.453 |
| COL27A1 | 0.949 | SETD8 | 0.818 | COL26A1 | 0.597 | TMEM38B | 0.522 | COL18A1 | 0.438 |
| COL11A1 | 0.946 | FKBP10 | 0.773 | COL9A3 | 0.56 | COL6A2 | 0.5 | COL20A1 | 0.427 |
| ADAMTS2 | 0.946 | NSD1 | 0.76 | COL9A2 | 0.554 | COL14A1 | 0.496 | COL17A1 | 0.413 |
| COL2A1 | 0.945 | CRTAP | 0.744 | KDM1A | 0.546 | COL8A2 | 0.491 | COL8A1 | 0.411 |
| COL5A3 | 0.934 | LEPRE1 | 0.701 | COL4A5 | 0.543 | COL6A1 | 0.488 | COL16A1 | 0.402 |

**3. Original images of western blots**

*PLOD1*-(HK-2-①, HK-2-②, HK-2-③, 786-O-①, 786-O-②,786-O-③, A498-①, A498-②, A498-③) for Fig. 2B


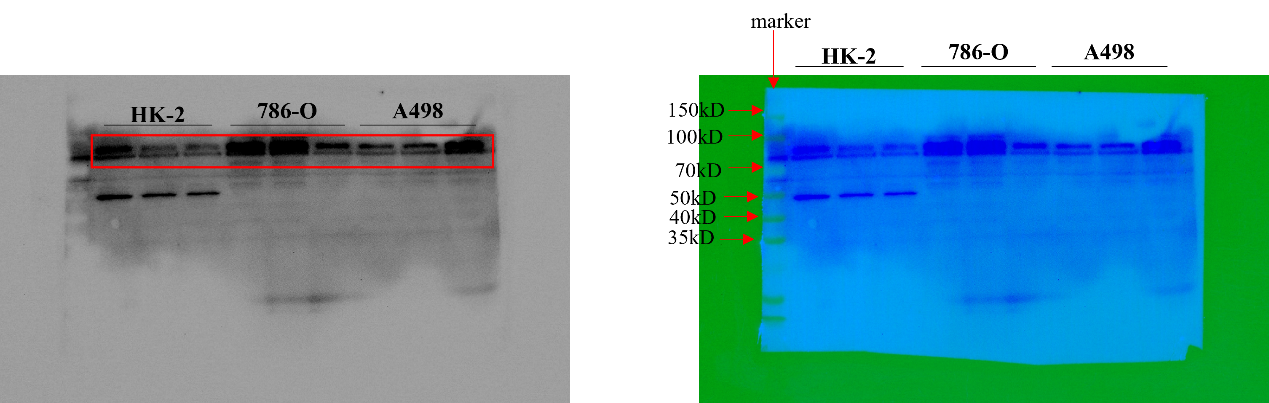


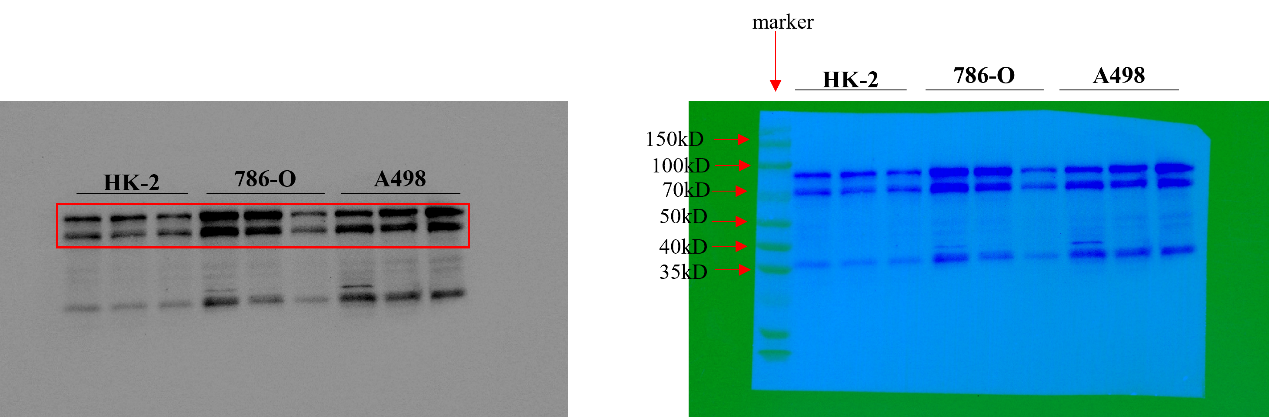
*PLOD2*-( HK-2-①, HK-2-②, HK-2-③, 786-O-①, 786-O-②,786-O-③, A498-①, A498-②, A498-③) for Fig. 2B


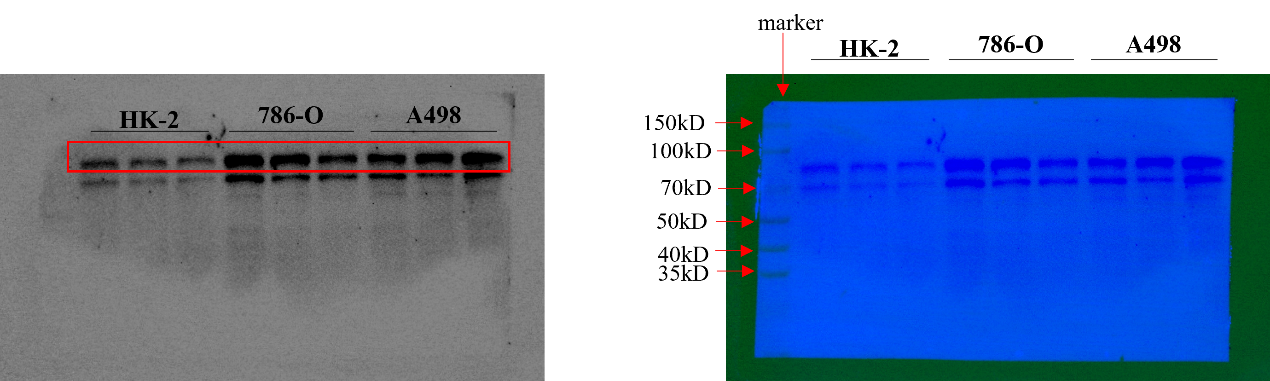
*PLOD3*-( HK-2-①, HK-2-②, HK-2-③, 786-O-①, 786-O-②,786-O-③, A498-①, A498-②, A498-③) for Fig. 2B


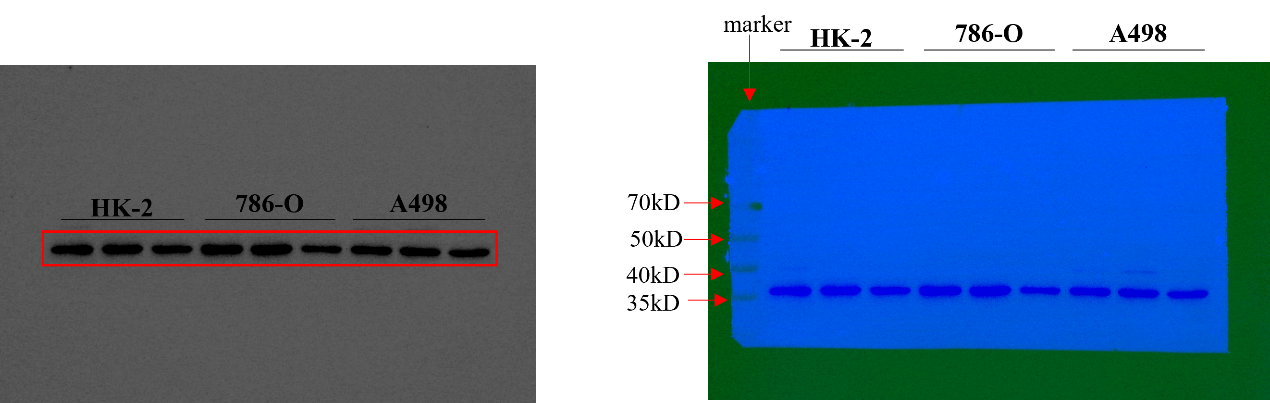
GAPDH-( HK-2-①, HK-2-②, HK-2-③, 786-O-①, 786-O-②,786-O-③, A498-①, A498-②, A498-③) for Fig. 2B


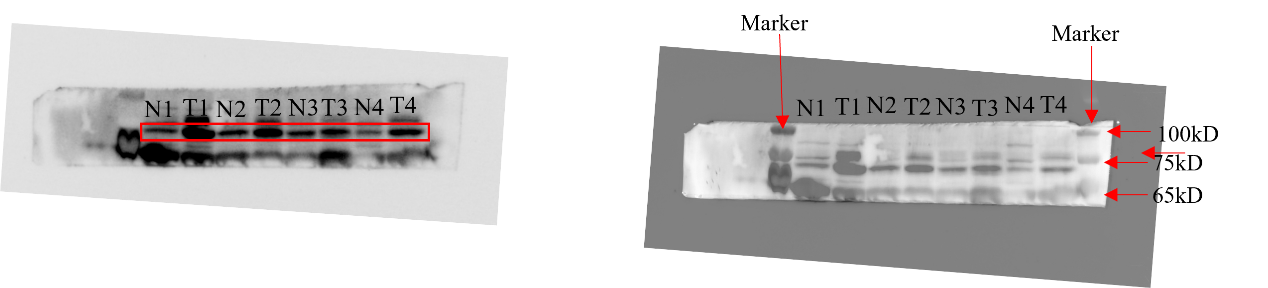
*PLOD1*-(N1, T1, N2, T2, N3, T3, N4, T4) for Fig. 2E


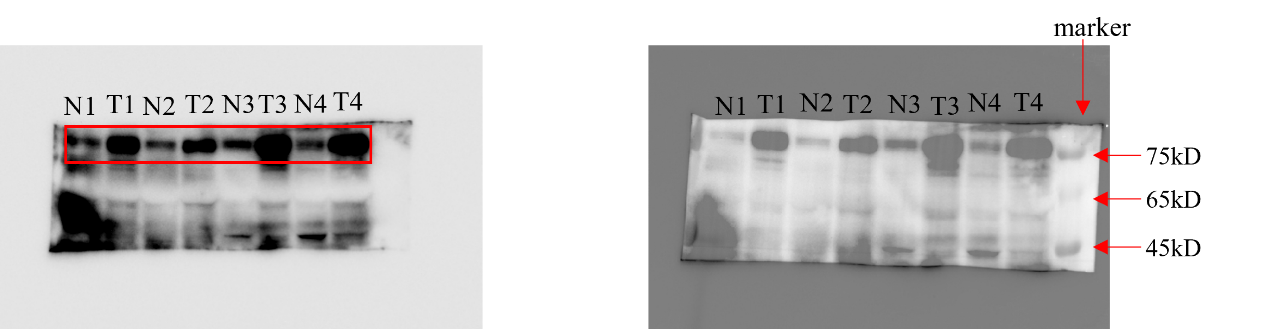
*PLOD2*-(N1, T1, N2, T2, N3, T3, N4, T4) for Fig. 2E


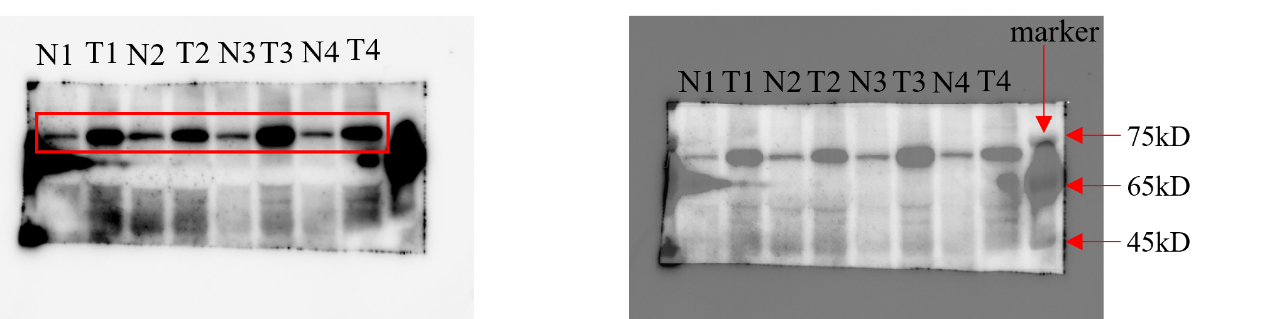
*PLOD3*-(N1, T1, N2, T2, N3, T3, N4, T4) for Fig. 2E


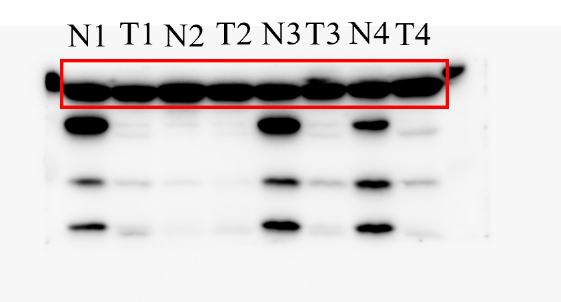
β-actin-(N1, T1, N2, T2, N3, T3, N4, T4) for Fig. 2E


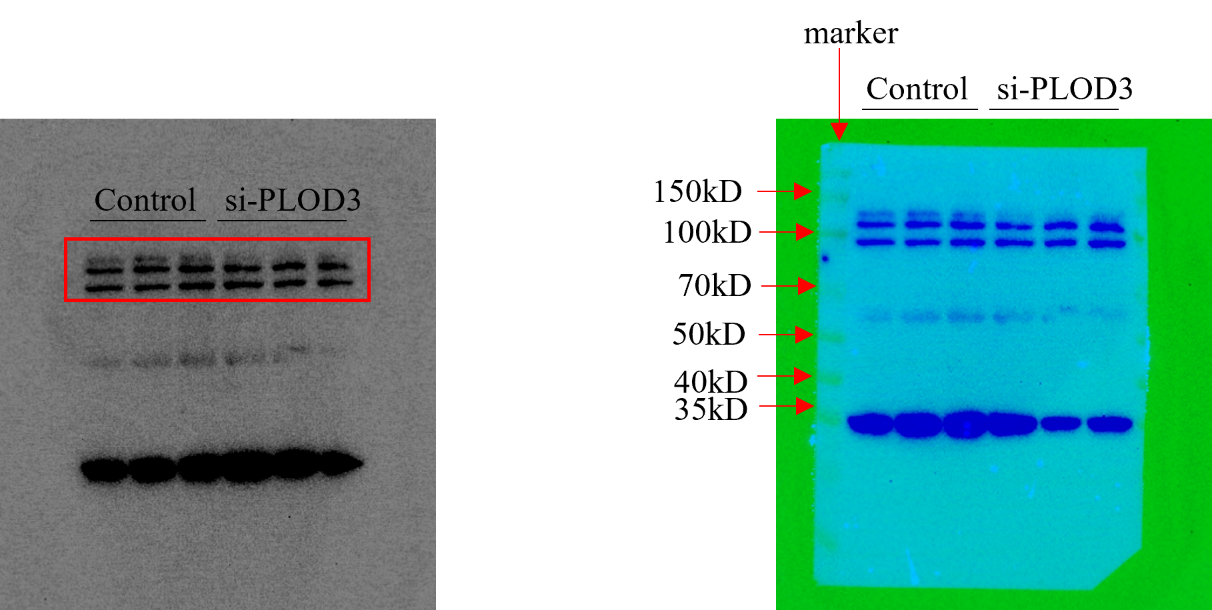
E-cadherin-(ctrl-1, ctrl-2, ctrl-3, si-*PLOD3*-1, si-*PLOD3*-2, si-*PLOD3*-3) for Fig. 8D


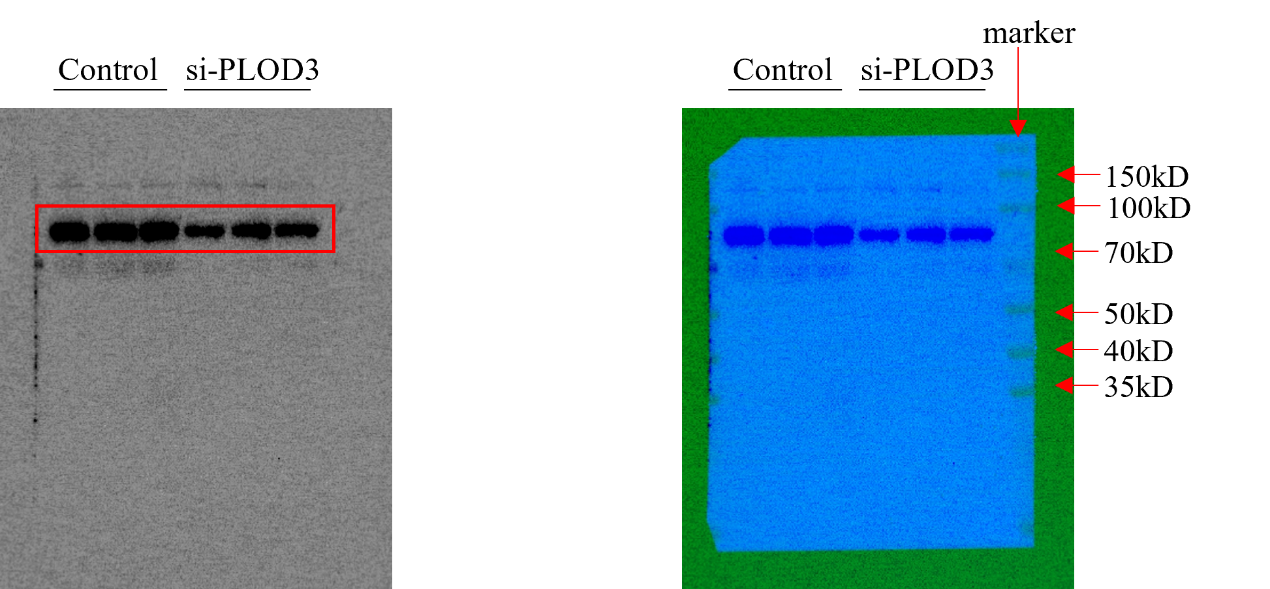
*PLOD3*-(ctrl-1, ctrl-2, ctrl-3, si-*PLOD3*-1, si-*PLOD3*-2, si-*PLOD3*-3) for Fig. 8D

Vimentin-(ctrl-1, ctrl-2, ctrl-3, si-*PLOD3*-1, si-*PLOD3*-2, si-*PLOD3*-3) for Fig. 8D


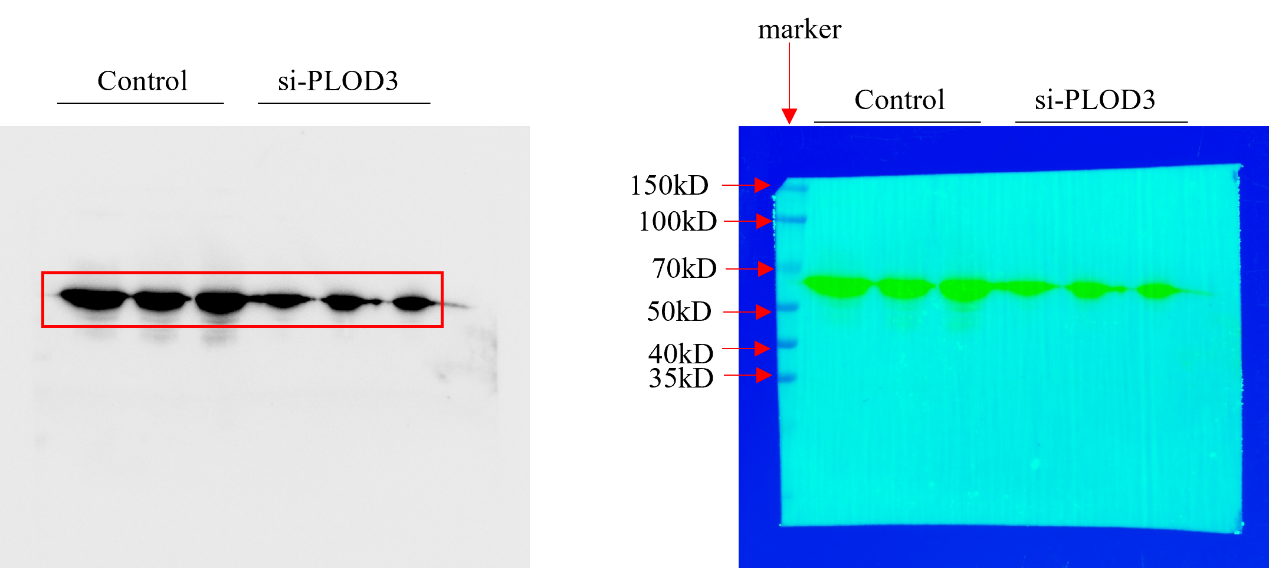


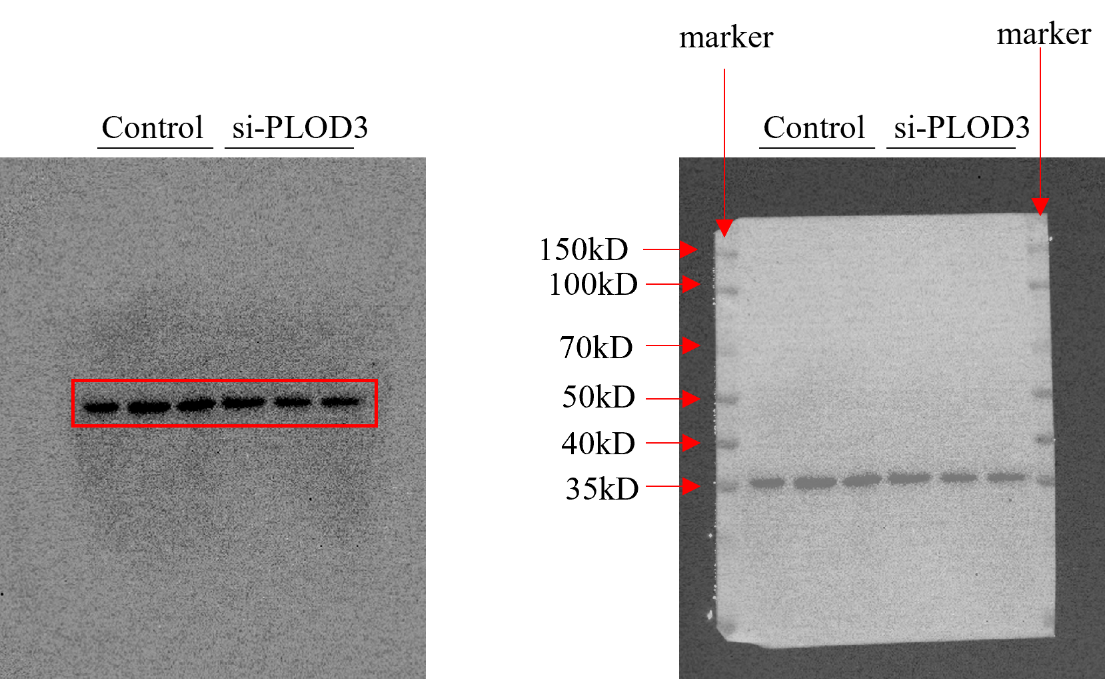
GAPDH-(ctrl-1, ctrl-2, ctrl-3, si-*PLOD3*-1, si-*PLOD3*-2, si-*PLOD3*-3) for Fig. 8D
